# Supplementary material for: Diagnosis of latent tuberculosis infection is associated with reduced HIV viral load and lower risk for opportunistic infections in people living with HIV
Source: PLoS Biol. 2020 Dec 7;18(12):e3000963. doi: 10.1371/journal.pbio.3000963 (PMC7721132; doi:10.1371/journal.pbio.3000963)
Supplement: S1 Text — (PDF) [file pbio.3000963.s001.pdf]

# Supplementary Information 1:

Diagnosis of latent tuberculosis infection is associated with reduced HIV viral load and lower risk for opportunistic infections in people living with HIV

Kusejko et al.

## Contents

1 Number and type of tuberculosis tests 2

2 Active tuberculosis 4

3 HIV set point viral load 6

4 Opportunistic infections 9

5 CD4 nadir 13

6 Ethnicity, region and HIV subtype 14

# 1 Number and type of tuberculosis tests

We analyzed information of 13326 tuberculin skin reactivity tests of 10649 patients performed between 1983 and 2019. Most tests were performed in the years 1992 – 1998 (see Figure A). After 2008, predominantly interferon-based screening tests were performed (see B). In total, we analyzed 3978 interferon-based screening tests of 3623 patients, the majority of the tests being quantiferon in-tube followed by liquid quantiferon tests.

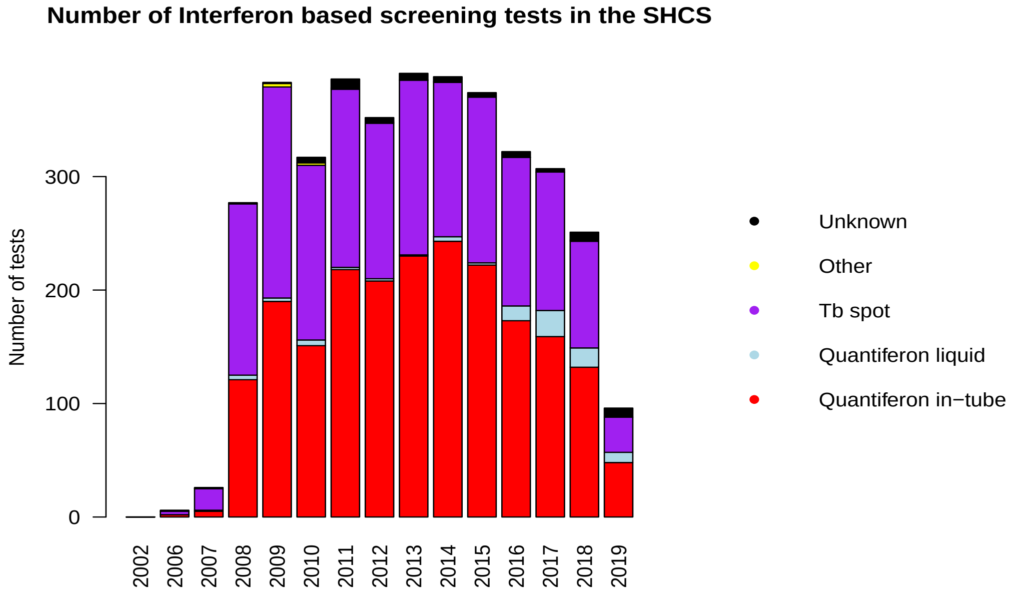


**Fig A.** Number of tuberculin skin reactivity tests in the SHCS (data source: A Data, FigureA1 Data)

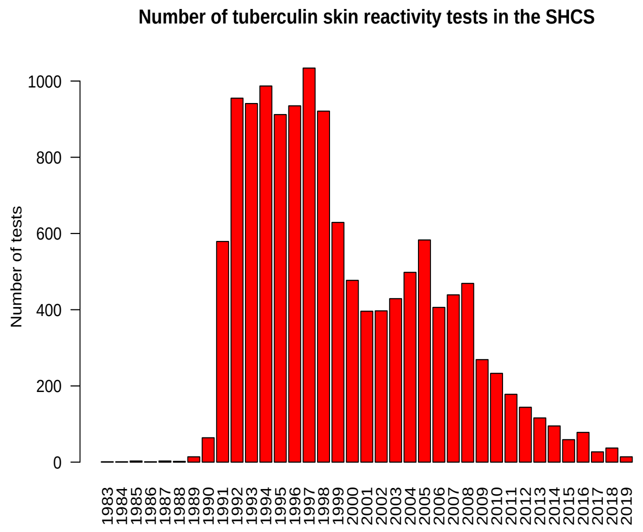


**Fig B.** Number of interferon based screening tests in the SHCS (data source: A Data, FigureA2 Data)

Of the 13675 patients with tuberculosis tests available, 11057 (80.1%) had only one test available. There were 187 patients with more than one tuberculosis test and different test results: 94 had at least one positive tuberculosis test followed by at least one negative tuberculosis test, 86 had at least one negative tuberculosis test followed by at least one positive tuberculosis test, and 7 patients had alternating results.

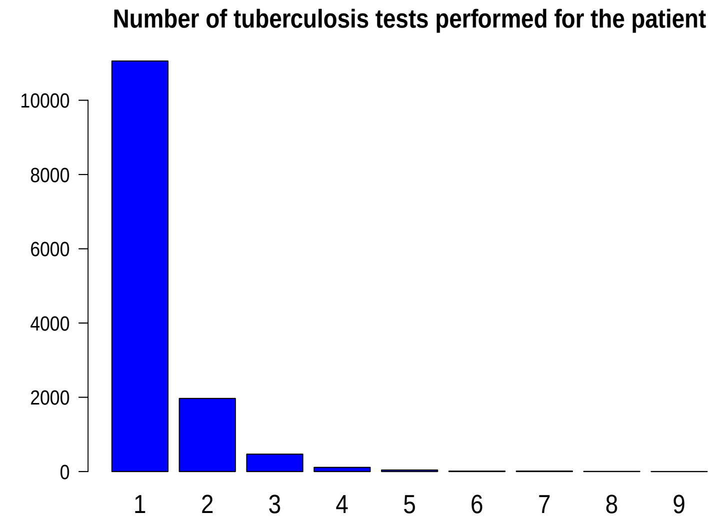


**Fig C.** Number of tuberculosis tests performed for the patients: The bars indicated the total number of patients who had 1, 2, etc tuberculosis tests in the SHCS (data source: A Data, FigureA3 Data)

Of the 17 243 tuberculosis test dates, 5750 (33.3%) tuberculosis tests were at date of the SHCS registration, 10 146 (58.8%) tests were within 1 year of the SHCS registration date, 182 (1.1%) more than 1 year before the SHCS registration and 6915 (40.1%) more than one year after SHCS registration.

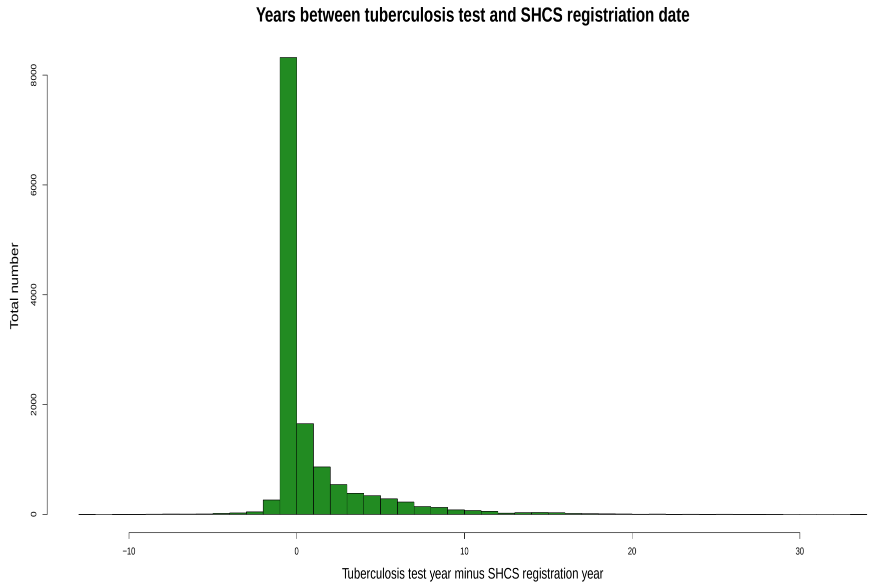


**Fig D.** Number of patients with a certain number of years between tuberculosis test year and SHCS registration year (data source: A Data, FigureA4 Data)

## 2 Active tuberculosis

Moreover, we looked at the cases of active tuberculosis in the SHCS. These were defined as follows (`http://www.shcs.ch/122-4-cdc-category-c-diagnoses`):

*Pulmonary tuberculosis* was defined by either of the following criteria:

- by culture (not PCR)
- by showing acid-fast bacilli in sputum that is not confirmed by culture or PCR and by responding to specific treatment
- suggestive infiltrate on CXR, anamnestic exposure to tuberculosis, or positive PPD test and response to specific treatment

*Extrapulmonary tuberculosis* was defined by either of the following criteria:

- by culture (not lung alone)
- by showing acid-fast bacilli in stool, blood, body fluid or tissue, or in histology of cervical or hilar lymph nodes, of a species not identified by culture, and there is a concurrent definitive diagnosis (by culture) of pulmonary tuberculosis
- responds to standard tuberculosis treatment

In total, 367 cases of extrapulmonary and 546 cases of pulmonary tuberculosis were reported in the SHCS (see Figure E).

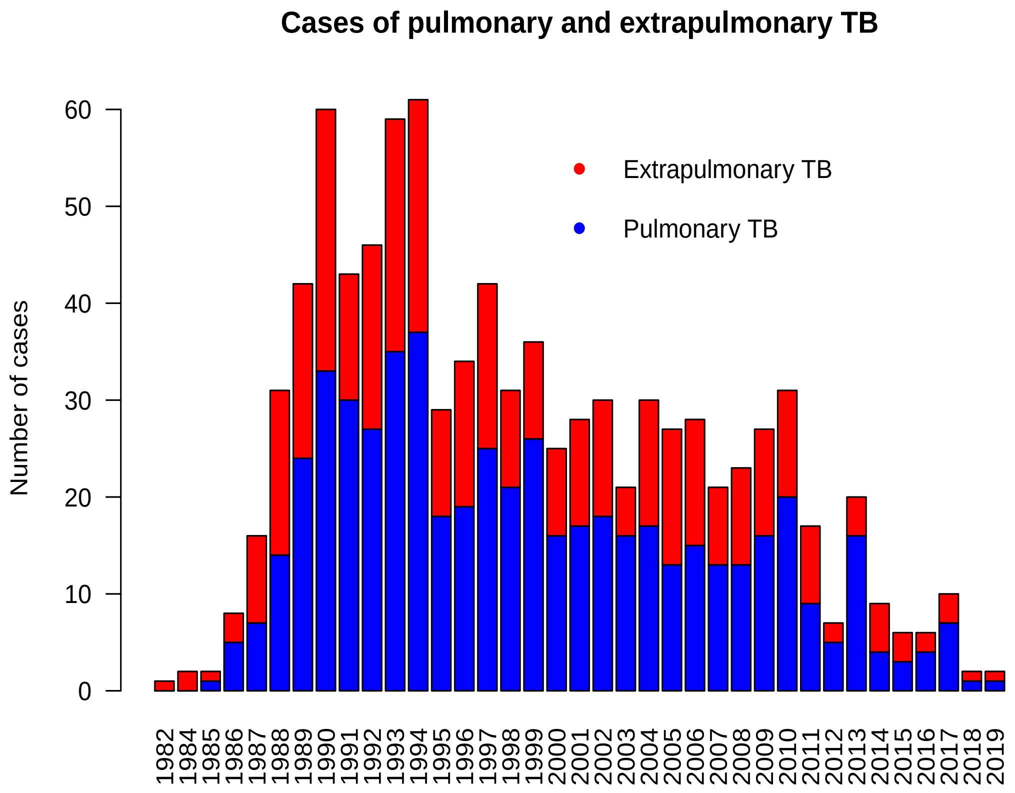


**Fig E.** Total cases of pulmonary and extrapulmonary tuberculosis cases reported in the SHCS (data source: A Data, FigureA5 Data)

# 3 HIV set point viral load

Of the 13943 patients included in our study population, the HIV set point viral load could be determined for 4516 patients. I.e., for these patients we had at least one ART-naive RNA measurement in the chronic phase of the HIV infection (at least 90 days after the first positive HIV test and before the occurrence of any opportunistic infection). The following table shows the basic characteristics of patients with available HIV set point virus load and those without. Of note, the diagnosis year of patients without SPVL measurement was on median 3 years earlier, 60% had an opportunistic infection and the CD4 nadir was very low with 140.

| Variable | | With SPVL | Without SPVL |
|---|---|---|---|
| Total (n) | | 4516 | 9427 |
| Sex | male (n, %) | 3204 (70.9%) | 6707 (71.1%) |
| | female (n, %) | 1312 (29.1%) | 2720 (28.9%) |
| Birth year (median, IQR) | | 1966 [1960,1973] | 1963 [1957,1971] |
| Ethnicity | white (n, %) | 3617 (80.1%) | 6505 (69%) |
| | black (n, %) | 519 (11.5%) | 1342 (14.2%) |
| | Hispano-American (n, %) | 149 (3.3%) | 257 (2.7%) |
| | Asian (n, %) | 135 (3%) | 323 (3.4%) |
| | unknown or other (n, %) | 94 (2.1%) | 996 (10.6%) |
| Region | Western Europe (n, %) | 3237 (71.7%) | 6221 (66%) |
| | Southern Europe (n, %) | 386 (8.5%) | 964 (10.2%) |
| | Eastern Africa (n, %) | 140 (3.1%) | 468 (5%) |
| | Middle Africa (n, %) | 161 (3.6%) | 407 (4.3%) |
| | South America (n, %) | 153 (3.4%) | 269 (2.9%) |
| | South-Eastern Asia (n, %) | 94 (2.1%) | 230 (2.4%) |
| | Eastern Europe (n, %) | 50 (1.1%) | 116 (1.2%) |
| | Other (n, %) | 295 (6.5%) | 752 (8%) |
| | Unknown (n, %) | 2 (0%) | 6 (0.1%) |
| HIV subtype | B (n, %) | 2846 (63%) | 3740 (39.7%) |
| | A (n, %) | 134 (3%) | 236 (2.5%) |
| | AG (n, %) | 149 (3.3%) | 182 (1.9%) |
| | AE (n, %) | 110 (2.4%) | 167 (1.8%) |
| | C (n, %) | 105 (2.3%) | 223 (2.4%) |
| | other (n, %) | 1431 (31.7%) | 5228 (55.5%) |
| Diagnosis year (median, IQR) | | 2000 [1992,2006] | 1997 [1990,2007] |
| Registration year (median, IQR) | | 2002 [1996,2008] | 1999 [1993,2010] |
| Transmission group | MSM (n, %) | 1729 (38.3%) | 3479 (36.9%) |
| | HET (n, %) | 1387 (30.7%) | 3331 (35.3%) |
| | IDU (n, %) | 1255 (27.8%) | 2137 (22.7%) |
| | other (n, %) | 145 (3.2%) | 480 (5.1%) |
| SHCS follow-up | active (n, %) | 2747 (60.8%) | 4981 (52.8%) |
| | lost to follow-up (n, %) | 1161 (25.7%) | 2037 (21.6%) |
| | death (n, %) | 608 (13.5%) | 2409 (25.6%) |
| | HIV/AIDS-related death (n, %) | 82 (1.8%) | 1183 (12.5%) |
| | Suicide (n, %) | 30 (0.7%) | 85 (0.9%) |
| | Overdose of narcotics (n, %) | 39 (0.9%) | 49 (0.5%) |
| | Accident (n, %) | 13 (0.3%) | 28 (0.3%) |
| | Other or unknown (n, %) | 444 (9.8%) | 1064 (11.3%) |
| First CD4 count (median, IQR) | | 460 [311.8,643] | 276 [120,470] |
| CD4 nadir (median, IQR) | | 230 [139,320] | 140 [37,280] |
| Primary infection (n, %) | | 259 (5.7%) | 639 (6.8%) |
| Opportunistic infection (n, %) | | 1800 (39.9%) | 5674 (60.2%) |

Moreover, the following two tables show the characteristics of LTBI and TB-uninfected patients with and

without available SPVL, respectively.

| Variable | | LTBI with SPVL | LTBI without SPVL |
|---|---|---|---|
| Total (n) | | 375 | 465 |
| Sex | male (n, %) | 261 (69.6%) | 288 (61.9%) |
| | female (n, %) | 114 (30.4%) | 177 (38.1%) |
| Birth year (median, IQR) | | 1968 [1962,1975] | 1969 [1961,1978] |
| Ethnicity | white (n, %) | 224 (59.7%) | 235 (50.5%) |
| | black (n, %) | 112 (29.9%) | 185 (39.8%) |
| | Hispano-American (n, %) | 15 (4%) | 12 (2.6%) |
| | Asian (n, %) | 17 (4.5%) | 28 (6%) |
| | unknown or other (n, %) | 7 (1.9%) | 5 (1.1%) |
| Region | Western Europe (n, %) | 189 (50.4%) | 185 (39.8%) |
| | Southern Europe (n, %) | 32 (8.5%) | 43 (9.2%) |
| | Eastern Africa (n, %) | 33 (8.8%) | 79 (17%) |
| | Middle Africa (n, %) | 36 (9.6%) | 57 (12.3%) |
| | South America (n, %) | 20 (5.3%) | 13 (2.8%) |
| | South-Eastern Asia (n, %) | 13 (3.5%) | 20 (4.3%) |
| | Eastern Europe (n, %) | 4 (1.1%) | 10 (2.2%) |
| | Other (n, %) | 48 (12.8%) | 58 (12.5%) |
| | Unknown (n, %) | 0 (0%) | 0 (0%) |
| HIV subtype | B (n, %) | 186 (49.6%) | 128 (27.5%) |
| | A (n, %) | 23 (6.1%) | 27 (5.8%) |
| | AG (n, %) | 19 (5.1%) | 16 (3.4%) |
| | AE (n, %) | 10 (2.7%) | 12 (2.6%) |
| | C (n, %) | 22 (5.9%) | 23 (4.9%) |
| | other (n, %) | 144 (38.4%) | 287 (61.7%) |
| Diagnosis year (median, IQR) | | 2003 [1997,2006] | 2003 [1997,2011] |
| Registration year (median, IQR) | | 2004 [1999,2008] | 2005 [1998,2013] |
| Transmission group | MSM (n, %) | 115 (30.7%) | 117 (25.2%) |
| | HET (n, %) | 153 (40.8%) | 253 (54.4%) |
| | IDU (n, %) | 88 (23.5%) | 60 (12.9%) |
| | other (n, %) | 19 (5.1%) | 35 (7.5%) |
| SHCS follow-up | active (n, %) | 216 (57.6%) | 298 (64.1%) |
| | lost to follow-up (n, %) | 112 (29.9%) | 136 (29.2%) |
| | death (n, %) | 47 (12.5%) | 31 (6.7%) |
| | HIV/AIDS-related death (n, %) | 4 (1.1%) | 4 (0.9%) |
| | Suicide (n, %) | 4 (1.1%) | 3 (0.6%) |
| | Overdose of narcotics (n, %) | 3 (0.8%) | 0 (0%) |
| | Accident (n, %) | 0 (0%) | 1 (0.2%) |
| | Other or unknown (n, %) | 36 (9.6%) | 23 (4.9%) |
| First CD4 count (median, IQR) | | 520 [390.5,719] | 383 [227,615] |
| CD4 nadir (median, IQR) | | 265 [195.5,363] | 264 [150,404] |
| Primary infection (n, %) | | 20 (5.3%) | 48 (10.3%) |
| Opportunistic infection (n, %) | | 85 (22.7%) | 152 (32.7%) |

| Variable | | No TB with SPVL | No TB without SPVL |
|---|---|---|---|
| Total (n) | | 4069 | 8264 |
| Sex | male (n, %) | 2896 (71.2%) | 5965 (72.2%) |
| | female (n, %) | 1173 (28.8%) | 2299 (27.8%) |
| Birth year (median, IQR) | | 1966 [1960,1972] | 1963 [1957,1971] |
| Ethnicity | white (n, %) | 3356 (82.5%) | 6008 (72.7%) |
| | black (n, %) | 382 (9.4%) | 949 (11.5%) |
| | Hispano-American (n, %) | 131 (3.2%) | 232 (2.8%) |
| | Asian (n, %) | 113 (2.8%) | 252 (3%) |
| | unknown or other (n, %) | 85 (2.1%) | 819 (9.9%) |
| Region | Western Europe (n, %) | 3014 (74.1%) | 5704 (69%) |
| | Southern Europe (n, %) | 348 (8.6%) | 859 (10.4%) |
| | Eastern Africa (n, %) | 99 (2.4%) | 315 (3.8%) |
| | Middle Africa (n, %) | 116 (2.9%) | 271 (3.3%) |
| | South America (n, %) | 129 (3.2%) | 230 (2.8%) |
| | South-Eastern Asia (n, %) | 79 (1.9%) | 173 (2.1%) |
| | Eastern Europe (n, %) | 46 (1.1%) | 97 (1.2%) |
| | Other (n, %) | 238 (5.8%) | 615 (7.4%) |
| | Unknown (n, %) | 1 (0%) | 4 (0%) |
| HIV subtype | B (n, %) | 2627 (64.6%) | 3452 (41.8%) |
| | A (n, %) | 106 (2.6%) | 180 (2.2%) |
| | AG (n, %) | 127 (3.1%) | 134 (1.6%) |
| | AE (n, %) | 98 (2.4%) | 138 (1.7%) |
| | C (n, %) | 75 (1.8%) | 164 (2%) |
| | other (n, %) | 1261 (31%) | 4468 (54.1%) |
| Diagnosis year (median, IQR) | | 1999 [1992,2006] | 1997 [1990,2007] |
| Registration year (median, IQR) | | 2002 [1996,2008] | 1999 [1993,2010] |
| Transmission group | MSM (n, %) | 1603 (39.4%) | 3230 (39.1%) |
| | HET (n, %) | 1199 (29.5%) | 2745 (33.2%) |
| | IDU (n, %) | 1144 (28.1%) | 1876 (22.7%) |
| | other (n, %) | 123 (3%) | 413 (5%) |
| SHCS follow-up | active (n, %) | 2490 (61.2%) | 4407 (53.3%) |
| | lost to follow-up (n, %) | 1031 (25.3%) | 1744 (21.1%) |
| | death (n, %) | 548 (13.5%) | 2113 (25.6%) |
| | HIV/AIDS-related death (n, %) | 74 (1.8%) | 989 (12%) |
| | Suicide (n, %) | 26 (0.6%) | 76 (0.9%) |
| | Overdose of narcotics (n, %) | 34 (0.8%) | 46 (0.6%) |
| | Accident (n, %) | 12 (0.3%) | 26 (0.3%) |
| | Other or unknown (n, %) | 402 (9.9%) | 976 (11.8%) |
| First CD4 count (median, IQR) | | 457 [306,637] | 278 [122,470] |
| CD4 nadir (median, IQR) | | 228 [135,317] | 140 [37,280] |
| Primary infection (n, %) | | 238 (5.8%) | 577 (7%) |
| Opportunistic infection (n, %) | | 1643 (40.4%) | 4824 (58.4%) |

# 4 Opportunistic infections

After excluding weight loss (unspecific disease), pulmonary tuberculosis and cervical dysplasia (as only women are at risk), the 10 most frequent opportunistic diseases diagnosed in our study population are: oral candidiasis, oral hairy leukoplakia, herpes zoster, candidiasis esophageal, pneumocystis pneumonia, HIV-related thrombocytopenia, Kaposi sarcoma, HIV-related encephalopathy, cerebral toxoplasmosis and bacterial pneumonia:

Table A: Frequency of opportunistic diseases in the study population

| | |
|---|---|
| Candidiasis oral | 3860 |
| Oral hairy leukoplakia | 1772 |
| Herpes zoster multidermatomal or relapse | 1553 |
| Candidiasis, oesophagial | 1289 |
| Pneumocystis pneumonia | 1261 |
| Thrombocytopenia, HIV-related | 894 |
| Weight loss | 642 |
| Kaposi sarcoma | 632 |
| Tuberculosis pulmonary | 546 |
| Cervical dysplasia | 476 |
| Encephalopathy, HIV-related | 452 |
| Toxoplasmosis, cerebral | 423 |
| Bacterial pneumonia, recurrent | 396 |
| Wasting Syndrome, AIDS-defining | 343 |
| Non-Hodgkin's lymphoma | 322 |
| M. avium - intracellulare, disseminated | 282 |
| CMV - retinitis | 259 |
| Diarrhea | 248 |
| Candidiasis vulvovaginal | 231 |
| CMV disease, other | 216 |
| Neuropathy, peripheral, HIV-related | 209 |
| Fever | 197 |
| Cryptosporidiosis, Diarrhoe > 1 month | 173 |
| Hodgkin's disease | 90 |
| M.other species diss.or extrapulm. | 76 |
| Hypertension, primary, pulmonary | 75 |
| Microsporidiosis | 74 |
| Cryptococcal meningitis | 63 |
| Herpes simplex disease, visceral | 60 |
| Cryptococcosis, other disseminated | 59 |
| Myelopathy, HIV-related | 59 |
| Intracerebral lesions, indeterminate | 44 |
| Mycobact. pulmonary disease, other | 38 |
| M. kansasii disease | 36 |
| Pneumonia, lymphoid, interstitial | 36 |
| Toxoplasma retinitis | 34 |
| Isosporiasis , Diarrhoe > 1 month | 33 |
| M.avium c.or kansasii (old code) | 29 |
| Carcinoma, cervical, invasive | 28 |
| M. genavense disease | 27 |
| Salmonella septicemia, recurrent | 21 |
| Aspergillosis | 20 |
| Leishmaniosis, visceral | 18 |
| Candidiasis of trachea, bronchi or lungs | 16 |
| Histoplasmosis disseminated | 15 |

| | |
|---|---|
| Pneumocystis disease, extrapulmonary | 8 |
| Pelvic inflammatory diseases | 7 |
| Toxoplasmosis disseminated | 7 |
| Angiomatosis, bacillary | 6 |
| Nocardiosis | 6 |
| Progressive outer retinal necrosis | 5 |
| Listeriosis | 3 |
| Pediatric category B disease | 3 |
| Rhodococcus equi disease | 3 |
| Extraintestinal strongyloidiasis | 2 |
| Coccidioidomycosis disseminated | 1 |

In 19708 (62.3%) cases, the diagnosis year of the opportunistic infection was in the same year or after the earliest tuberculosis test of the patient (see Figure F).

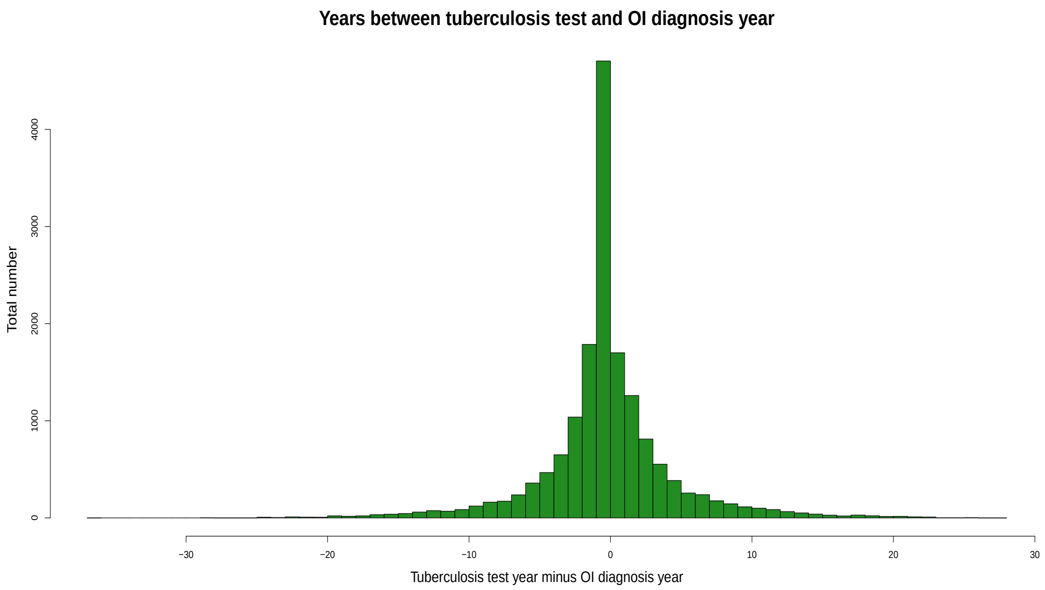


**Fig F.** Numbers of patients with a certain number of years between tuberculosis test and OI diagnosis year (data source: A Data, FigureA6 Data)

In particular, both the tuberculosis test and the diagnosis of the OI were clustered around the registration date of the SHCS, i.e., in most cases shortly after HIV diagnosis (see Figure G).

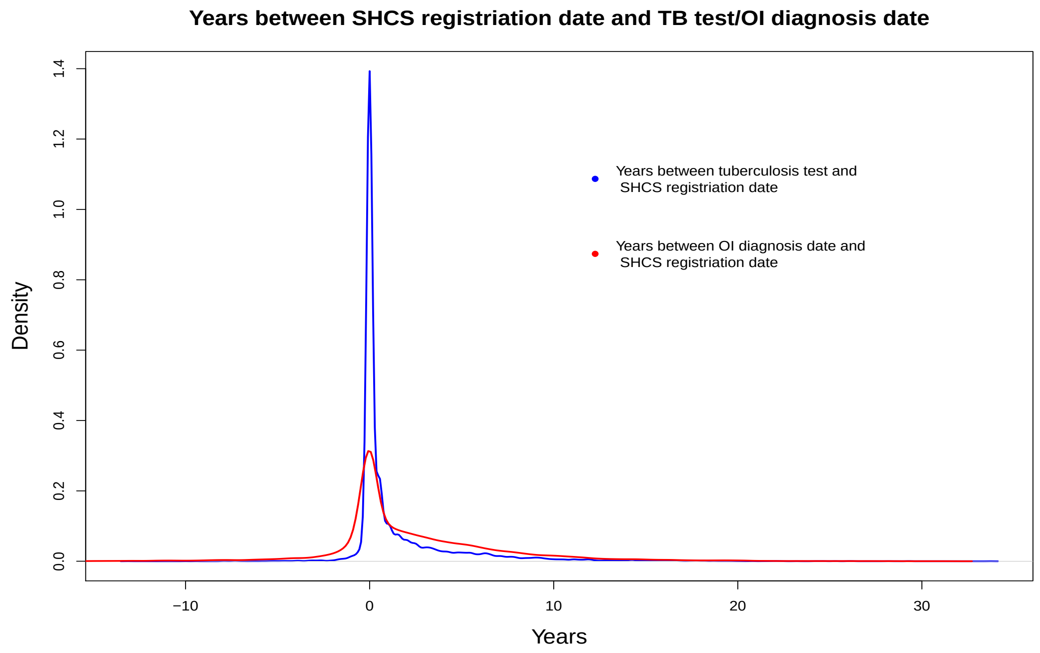


**Fig G.** Timing of the tuberculosis test and OI diagnosis with respect to the SHCS registration time (data source: A Data, FigureA7 Data)

### Timepoint of the candida stomatitis, oral hairy leukoplakia and herpes zoster diagnosis

Moreover, 2190 (59.6%) cases of candida stomatitis, 1166 (67.1%) cases of oral hairy leukoplakia and 913 (60.1%) cases of herpes zoster were diagnosed in the same year or after the tuberculosis test. (see Figures H, I, J)

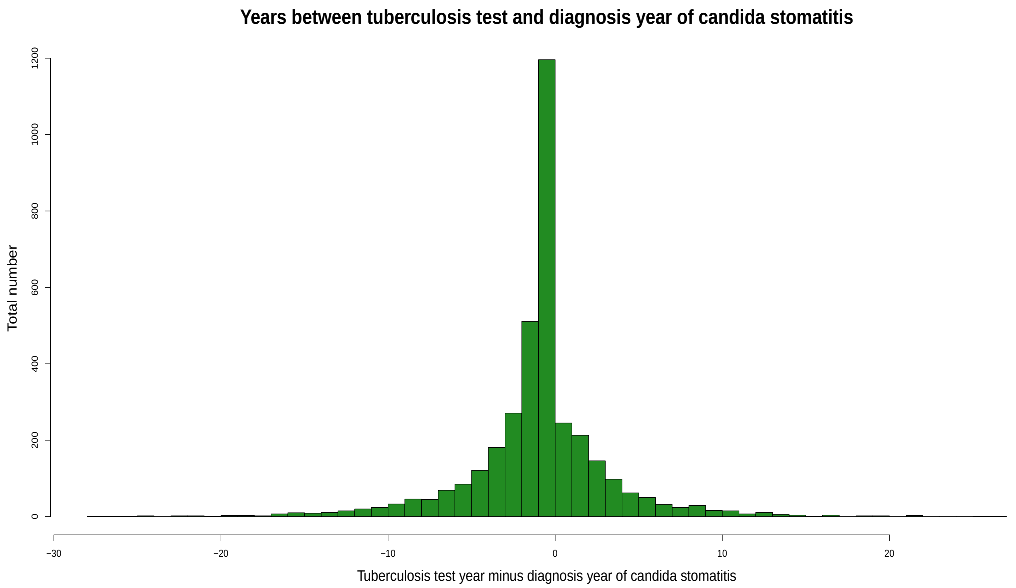


**Fig H.** Years between tuberculosis test and diagnosis year of candida stomatitis (data source: A Data, FigureA8 Data)

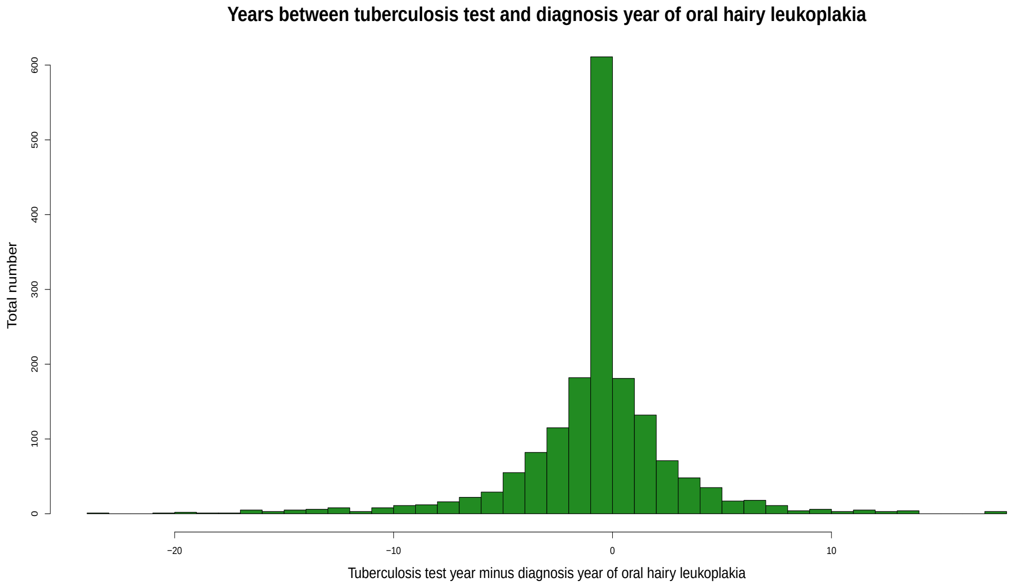


**Fig I.** Years between tuberculosis test and diagnosis year of oral hairy leukoplakia (data source: A Data, FigureA9 Data)

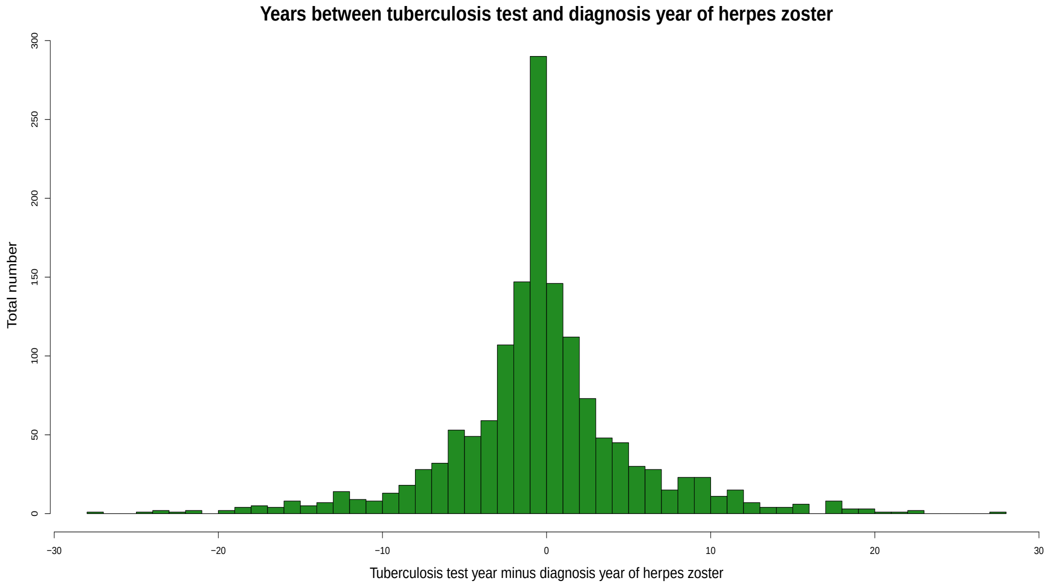


**Fig J.** Years between tuberculosis test and diagnosis year of herpes zoster (data source: A Data, FigureA10 Data)

# 5 CD4 nadir

The following Figure illustrates that the time point of the CD4 nadir measurements was close to the tuberculosis test for most patients.

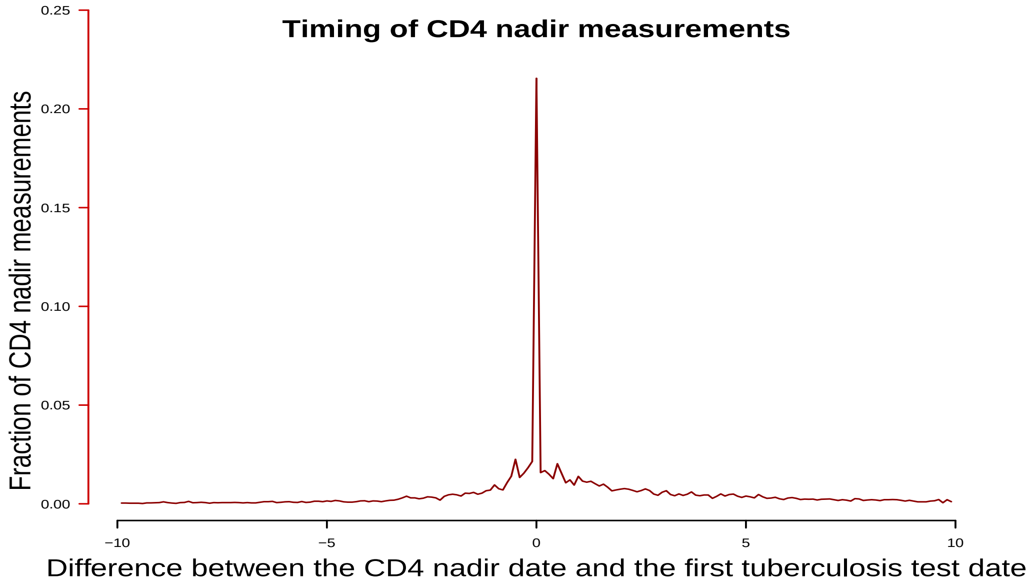


**Fig K.** Years between the CD4 nadir test date and the date of the first tuberculosis test (data source: A Data, FigureA11 Data)

# 6 Ethnicity, region and HIV subtype

As extension for Table 1 in the main manuscript, the following table shows the ethnicity, region and HIV subtype of the three groups: LTBI, active TB and TB uninfected.

| Variable | | no TB | LTBI | active TB |
|---|---|---|---|---|
| Total (n) | | 12333 | 840 | 770 |
| Ethnicity | white (n, %) | 9364 (75.9%) | 459 (54.6%) | 299 (38.8%) |
| | black (n, %) | 1331 (10.8%) | 297 (35.4%) | 233 (30.3%) |
| | Hispano-American (n, %) | 363 (2.9%) | 27 (3.2%) | 16 (2.1%) |
| | Asian (n, %) | 365 (3%) | 45 (5.4%) | 48 (6.2%) |
| | unknown or other (n, %) | 904 (7.3%) | 12 (1.4%) | 174 (22.6%) |
| Region | Western Europe (n, %) | 8718 (70.7%) | 374 (44.5%) | 366 (47.5%) |
| | Southern Europe (n, %) | 1207 (9.8%) | 75 (8.9%) | 68 (8.8%) |
| | Eastern Africa (n, %) | 414 (3.4%) | 112 (13.3%) | 82 (10.6%) |
| | Middle Africa (n, %) | 387 (3.1%) | 93 (11.1%) | 88 (11.4%) |
| | South America (n, %) | 359 (2.9%) | 33 (3.9%) | 30 (3.9%) |
| | South-Eastern Asia (n, %) | 252 (2%) | 33 (3.9%) | 39 (5.1%) |
| | Eastern Europe (n, %) | 143 (1.2%) | 14 (1.7%) | 9 (1.2%) |
| | Other (n, %) | 853 (6.9%) | 106 (12.6%) | 88 (11.4%) |
| | Unknown (n, %) | 5 (0%) | 0 (0%) | 3 (0.4%) |
| HIV subtype | B (n, %) | 6079 (49.3%) | 314 (37.4%) | 193 (25.1%) |
| | A (n, %) | 286 (2.3%) | 50 (6%) | 34 (4.4%) |
| | AG (n, %) | 261 (2.1%) | 35 (4.2%) | 35 (4.5%) |
| | AE (n, %) | 236 (1.9%) | 22 (2.6%) | 19 (2.5%) |
| | C (n, %) | 239 (1.9%) | 45 (5.4%) | 44 (5.7%) |
| | other (n, %) | 5729 (46.5%) | 431 (51.3%) | 499 (64.8%) |
