# Supplementary material for: Diagnosis of latent tuberculosis infection is associated with reduced HIV viral load and lower risk for opportunistic infections in people living with HIV
Source: PLoS Biol. 2020 Dec 7;18(12):e3000963. doi: 10.1371/journal.pbio.3000963 (PMC7721132; doi:10.1371/journal.pbio.3000963)
Supplement: S2 Text — (PDF) [file pbio.3000963.s002.pdf]

Supplementary Information 2:

Diagnosis of latent tuberculosis infection is associated with reduced HIV viral load and lower risk for opportunistic infections in people living with HIV

Kusejko et al.

Contents

1 Specificity of the tuberculosis test **2**
1.1 All tested patients . . . 2
1.2 Ambiguous test results . . . 4
1.3 CD4 cell count and tuberculosis test . . . 7

2 Timing of the tuberculosis test **13**
2.1 Tuberculosis test within 1 year of SHCS registration . . . 13
2.2 Timing of opportunistic infections . . . 15

3 Antiretroviral treatment **18**

4 Prophylactic tuberculosis treatment **20**

5 Ethnicity, region and HIV subtype **23**

6 Pooling latent and active tuberculosis **32**

7 Alternative definitions for HIV set point virus load **35**

8 Summary of the sensitivity analyses **36**
8.1 Set point virus load . . . 36
8.2 Candida stomatitis . . . 36
8.3 Oral hairy leukoplakia . . . 37
8.4 Herpes zoster . . . 37

1 Specificity of the tuberculosis test

1.1 All tested patients

In the original analysis, we excluded patients with different tuberculosis test results over time. Including these 187 patients again yields a study population of 14130 patients, 12333 tuberculosis-uninfected patients, 1027 with latent tuberculosis infection and 770 with active tuberculosis.

A) HIV set point virus load

	Original results	Including all patients with at least one tuberculosis test
Sample size	4516	4605
Overall mean:	4.4	4.4
Mean: No tuberculosis	4.43	4.43
Mean: LTBI	4.11	4.14
Mean: Active MTB infection	4.63	4.63
unadjusted: LTBI	-0.32 [-0.4, -0.24]	-0.29 [-0.36, -0.21]
unadjusted: active MTB infection	0.2 [0.03, 0.37]	0.2 [0.03, 0.37]
adjusted: LTBI	-0.21 [-0.28, -0.13]	-0.19 [-0.26, -0.12]
adjusted: active MTB infection	0.14 [-0.02, 0.31]	0.14 [-0.02, 0.31]

B) Opportunistic infections

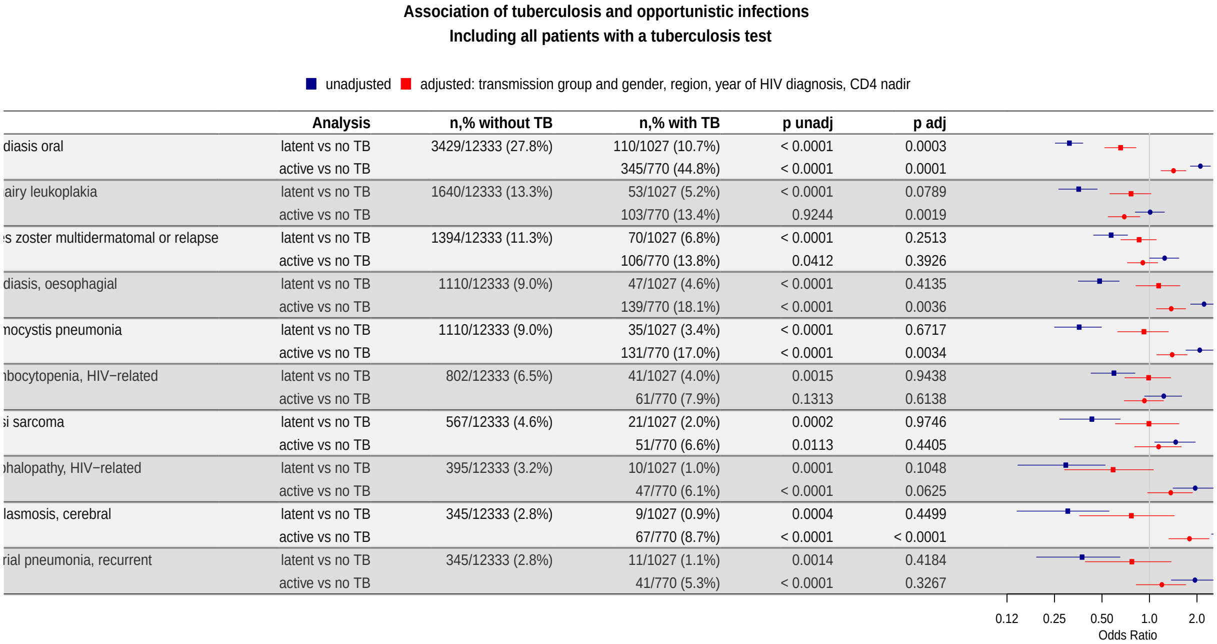

	Analysis	n,% without TB	n,% with TB	p unadj	p adj
diasis oral	latent vs no TB	3429/12333 (27.8%)	110/1027 (10.7%)	< 0.0001	0.0003
	active vs no TB		345/770 (44.8%)	< 0.0001	0.0001
airy leukoplakia	latent vs no TB	1640/12333 (13.3%)	53/1027 (5.2%)	< 0.0001	0.0789
	active vs no TB		103/770 (13.4%)	0.9244	0.0019
s zoster multidermatomal or relapse	latent vs no TB	1394/12333 (11.3%)	70/1027 (6.8%)	< 0.0001	0.2513
	active vs no TB		106/770 (13.8%)	0.0412	0.3926
diasis, oesophagial	latent vs no TB	1110/12333 (9.0%)	47/1027 (4.6%)	< 0.0001	0.4135
	active vs no TB		139/770 (18.1%)	< 0.0001	0.0036
nocystis pneumonia	latent vs no TB	1110/12333 (9.0%)	35/1027 (3.4%)	< 0.0001	0.6717
	active vs no TB		131/770 (17.0%)	< 0.0001	0.0034
ıbocytopenia, HIV−related	latent vs no TB	802/12333 (6.5%)	41/1027 (4.0%)	0.0015	0.9438
	active vs no TB		61/770 (7.9%)	0.1313	0.6138
si sarcoma	latent vs no TB	567/12333 (4.6%)	21/1027 (2.0%)	0.0002	0.9746
	active vs no TB		51/770 (6.6%)	0.0113	0.4405
ıhalopathy, HIV−related	latent vs no TB	395/12333 (3.2%)	10/1027 (1.0%)	0.0001	0.1048
	active vs no TB		47/770 (6.1%)	< 0.0001	0.0625
lasmosis, cerebral	latent vs no TB	345/12333 (2.8%)	9/1027 (0.9%)	0.0004	0.4499
	active vs no TB		67/770 (8.7%)	< 0.0001	< 0.0001
rial pneumonia, recurrent	latent vs no TB	345/12333 (2.8%)	11/1027 (1.1%)	0.0014	0.4184
	active vs no TB		41/770 (5.3%)	< 0.0001	0.3267

Fig A. Association of the ten most frequent opportunistic infections with tuberculosis (TB) infection: Patients with active MTB infection and latent tuberculosis infection (LTBI) compared to tuberculosis uninfected patients, respectively (active vs no TB, latent vs no TB)

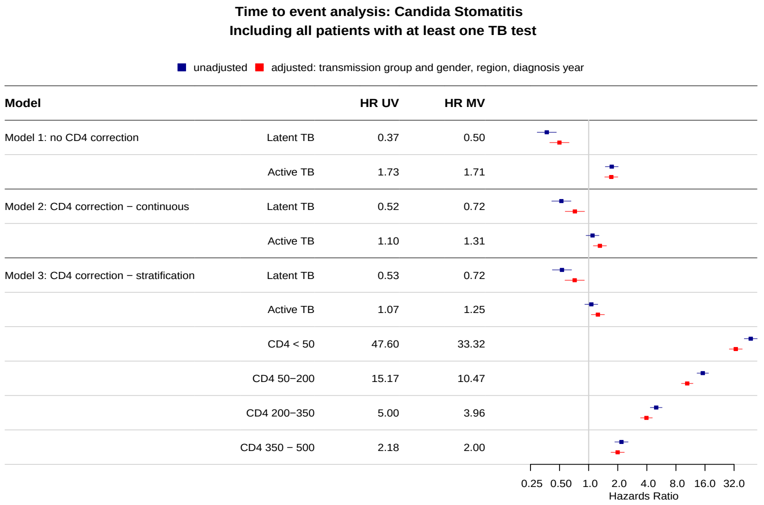

Fig B. Time to event analysis of the occurrence of candida stomatitis: Patients with active MTB infection or latent MTB infection compared to tuberculosis uninfected patients, respectively.

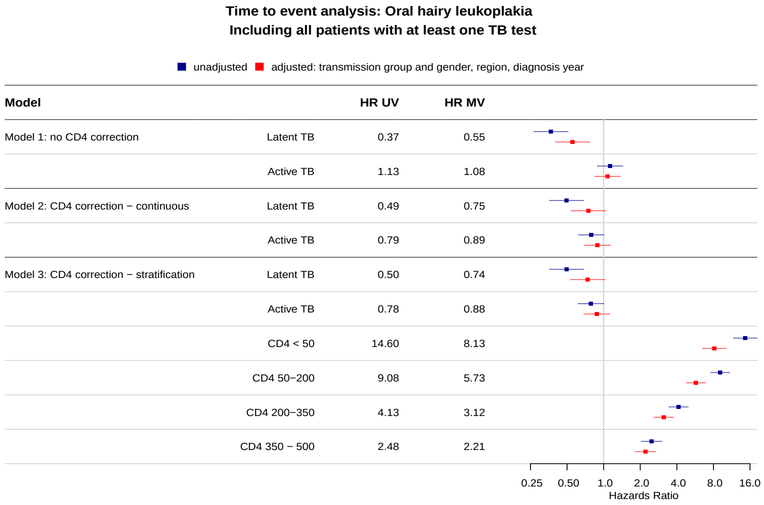

Fig C. Time to event analysis of the occurrence of oral hairy leukoplakia: Patients with active MTB infection or latent MTB infection compared to tuberculosis uninfected patients, respectively.

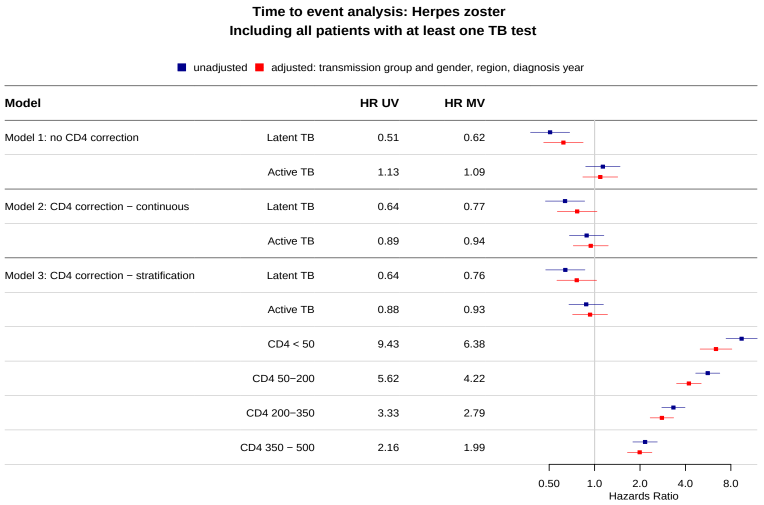

Fig D. Time to event analysis of the occurrence of herpes zoster: Patients with active MTB infection or latent MTB infection compared to tuberculosis uninfected patients, respectively.

1.2 Ambiguous test results

We excluded patients with borderline test results or different results in the two types of tuberculosis test, i.e., positive for the skin reactivity test and negative for the interferon-based test, or vice versa. In total, 95 patients had ambiguous test results, 83 were in the category of tuberculosis uninfected patients and 12 in the group of latently infected patients. Excluding these 95 patients from the original study population yield the following results.

A) HIV set point virus load

	Original results	Excluding patients with ambiguous results
Sample size	4516	4483
Overall mean:	4.4	4.41
Mean: No tuberculosis	4.43	4.43
Mean: LTBI	4.11	4.11
Mean: Active MTB infection	4.63	4.63
unadjusted: LTBI	-0.32 [-0.4, -0.24]	-0.32 [-0.4, -0.24]
unadjusted: active MTB infection	0.2 [0.03, 0.37]	0.2 [0.02, 0.37]
adjusted: LTBI	-0.21 [-0.28, -0.13]	-0.22 [-0.29, -0.14]
adjusted: active MTB infection	0.14 [-0.02, 0.31]	0.14 [-0.03, 0.3]

B) Opportunistic infections

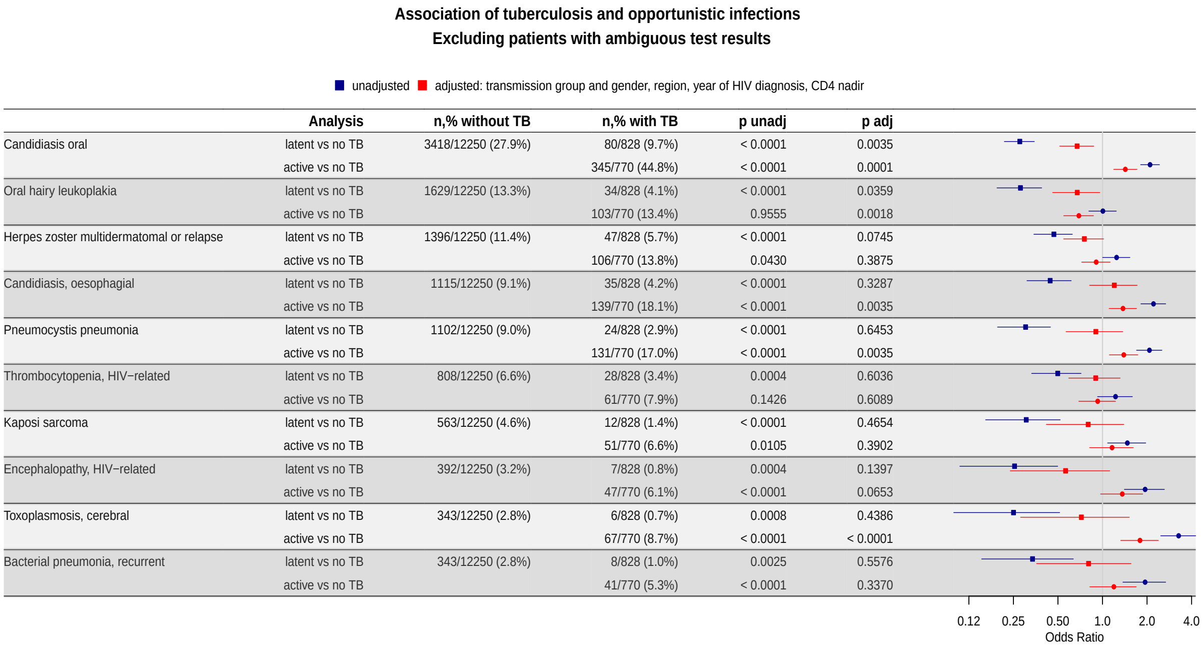

	Analysis	n,% without TB	n,% with TB	p unadj	p adj
Candidiasis oral	latent vs no TB	3418/12250 (27.9%)	80/828 (9.7%)	< 0.0001	0.0035
	active vs no TB		345/770 (44.8%)	< 0.0001	0.0001
Oral hairy leukoplakia	latent vs no TB	1629/12250 (13.3%)	34/828 (4.1%)	< 0.0001	0.0359
	active vs no TB		103/770 (13.4%)	0.9555	0.0018
Herpes zoster multidermatomal or relapse	latent vs no TB	1396/12250 (11.4%)	47/828 (5.7%)	< 0.0001	0.0745
	active vs no TB		106/770 (13.8%)	0.0430	0.3875
Candidiasis, oesophagial	latent vs no TB	1115/12250 (9.1%)	35/828 (4.2%)	< 0.0001	0.3287
	active vs no TB		139/770 (18.1%)	< 0.0001	0.0035
Pneumocystis pneumonia	latent vs no TB	1102/12250 (9.0%)	24/828 (2.9%)	< 0.0001	0.6453
	active vs no TB		131/770 (17.0%)	< 0.0001	0.0035
Thrombocytopenia, HIV−related	latent vs no TB	808/12250 (6.6%)	28/828 (3.4%)	0.0004	0.6036
	active vs no TB		61/770 (7.9%)	0.1426	0.6089
Kaposi sarcoma	latent vs no TB	563/12250 (4.6%)	12/828 (1.4%)	< 0.0001	0.4654
	active vs no TB		51/770 (6.6%)	0.0105	0.3902
Encephalopathy, HIV−related	latent vs no TB	392/12250 (3.2%)	7/828 (0.8%)	0.0004	0.1397
	active vs no TB		47/770 (6.1%)	< 0.0001	0.0653
Toxoplasmosis, cerebral	latent vs no TB	343/12250 (2.8%)	6/828 (0.7%)	0.0008	0.4386
	active vs no TB		67/770 (8.7%)	< 0.0001	< 0.0001
Bacterial pneumonia, recurrent	latent vs no TB	343/12250 (2.8%)	8/828 (1.0%)	0.0025	0.5576
	active vs no TB		41/770 (5.3%)	< 0.0001	0.3370

Fig E. Association of the ten most frequent opportunistic infections with tuberculosis (TB) infection: Patients with active MTB infection and latent tuberculosis infection (LTBI) compared to tuberculosis uninfected patients, respectively (active vs no TB, latent vs no TB)

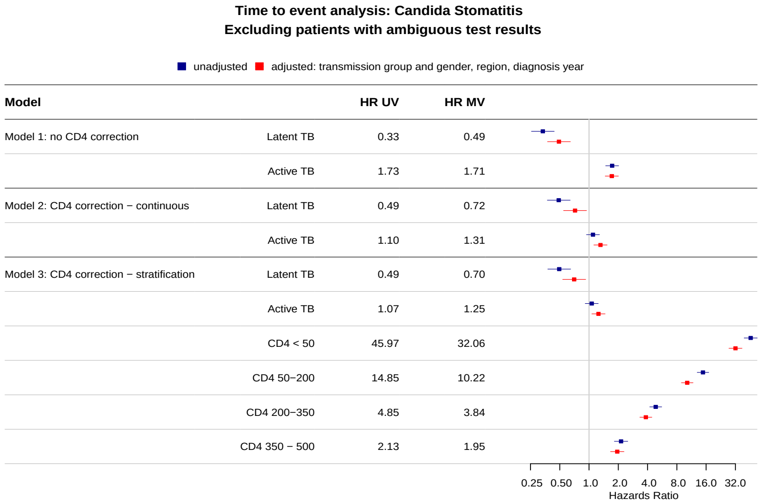

Model		HR UV	HR MV
Model 1: no CD4 correction	Latent TB	0.33	0.49
	Active TB	1.73	1.71
Model 2: CD4 correction − continuous	Latent TB	0.49	0.72
	Active TB	1.10	1.31
Model 3: CD4 correction − stratification	Latent TB	0.49	0.70
	Active TB	1.07	1.25
	CD4 < 50	45.97	32.06
	CD4 50−200	14.85	10.22
	CD4 200−350	4.85	3.84
	CD4 350 − 500	2.13	1.95

Fig F. Time to event analysis of the occurrence of candida stomatitis: Patients with active MTB infection or latent MTB infection compared to tuberculosis uninfected patients, respectively.

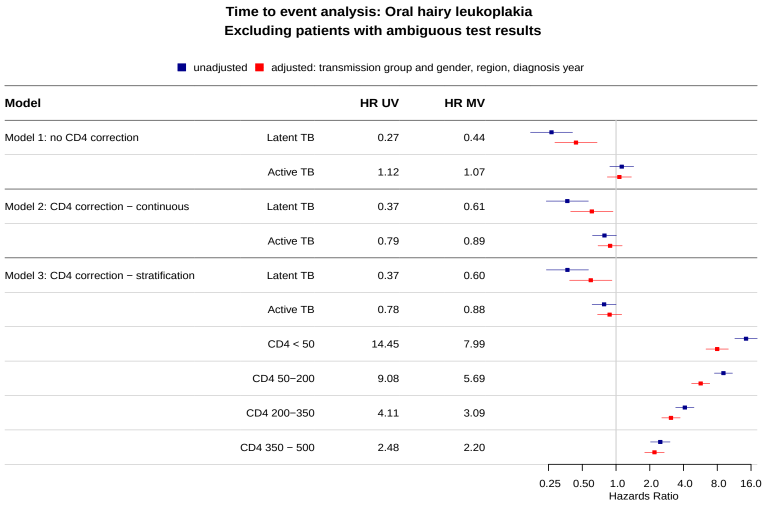

Fig G. Time to event analysis of the occurrence of oral hairy leukoplakia: Patients with active MTB infection or latent MTB infection compared to tuberculosis uninfected patients, respectively.

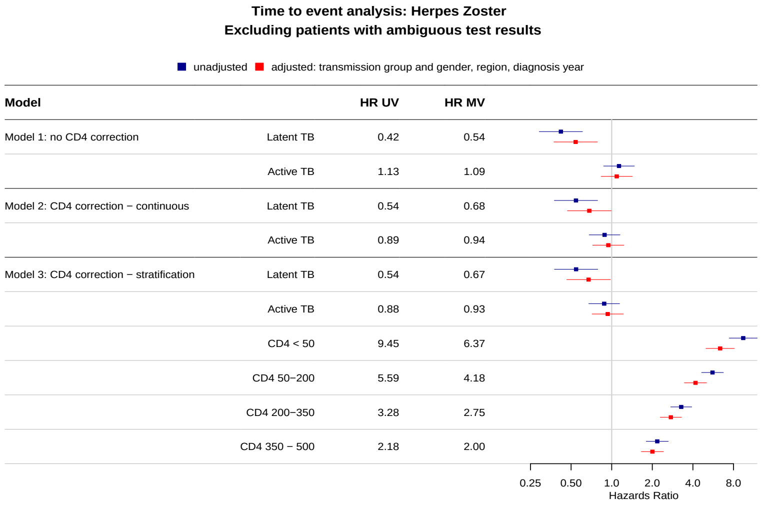

Fig H. Time to event analysis of the occurrence of herpes zoster: Patients with active MTB infection or latent MTB infection compared to tuberculosis uninfected patients, respectively.

1.3 CD4 cell count and tuberculosis test

It was observed that the probability of false negative tuberculosis tests is higher in immunosuppressed patients. To assess the impact of the immune status at the tuberculosis test date, we performed the following sensitivity analyses:

1. Restricting the study population to patients with a CD4 count of $>$ 350 cells/mL at tuberculosis test date. Tuberculosis was defined using only test results where the patient had $>$ 350 CD4 cells/mL.
2. Restricting the study population to patients with a CD4 count of $>$ 500 cells/mL at tuberculosis test date. Tuberculosis was defined using only test results where the patient had $>$ 500 CD4 cells/mL.
3. Using the original study population, we corrected for the CD4 count at the time the first tuberculosis test was taken.

While CD4 cell counts were available for 97.5% most tuberculosis test dates, this was not the case for HIV RNA values. In particular, no HIV RNA measurements were available for the first years (see Figure I):

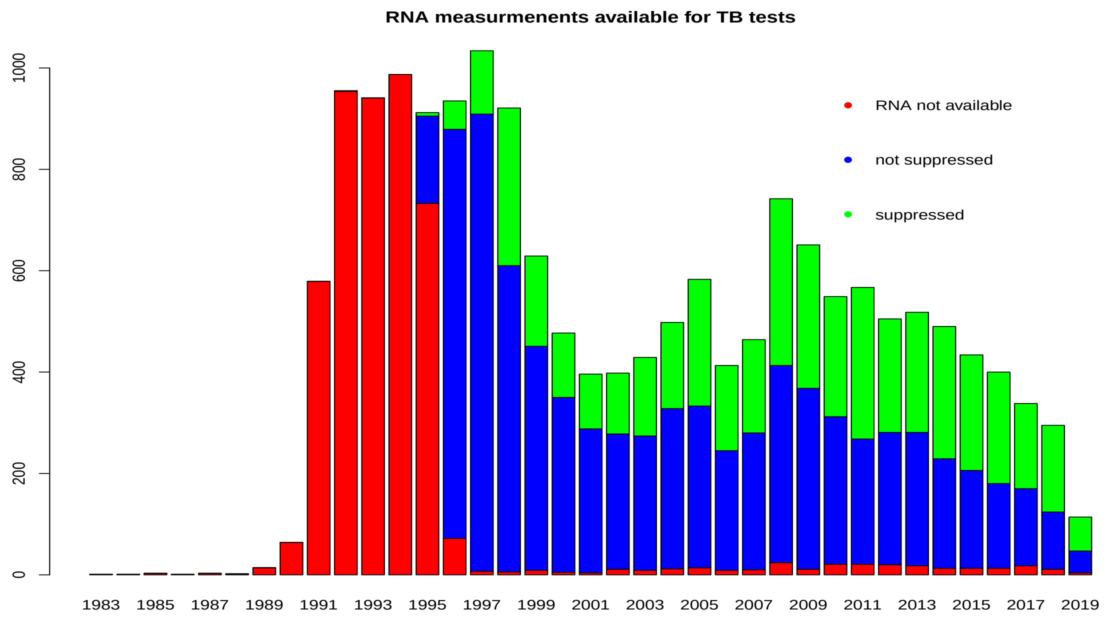

Fig I. Availability of RNA measurements at the tuberculosis test date and number of suppressed patients.

A) HIV set point virus load

	Original results	> 350 CD4	> 500 CD4	Correcting for CD4 at TB test date
Sample size	4516	3082	1948	4516
Overall mean:	4.4	4.31	4.27	4.4
Mean: No tuberculosis	4.43	4.34	4.29	4.43
Mean: LTBI	4.11	4.03	4	4.11
Mean: Active MTB infection	4.63	4.63	4.63	4.63
unadjusted: LTBI	-0.32 [-0.4, -0.24]	-0.3 [-0.39, -0.22]	-0.29 [-0.4, -0.19]	-0.32 [-0.4, -0.24]
unadjusted: active MTB infection	0.2 [0.03, 0.37]	0.29 [0.12, 0.46]	0.33 [0.16, 0.51]	0.2 [0.03, 0.37]
adjusted: LTBI	-0.21 [-0.28, -0.13]	-0.23 [-0.32, -0.15]	-0.24 [-0.34, -0.14]	-0.21 [-0.28, -0.13]
adjusted: active MTB infection	0.14 [-0.02, 0.31]	0.2 [0.03, 0.37]	0.21 [0.04, 0.38]	0.16 [-0.04, 0.36]

By design, we cannot correct for the HIV RNA at TB test date, as this would be a correction of the outcome variable.

B) Opportunistic infections

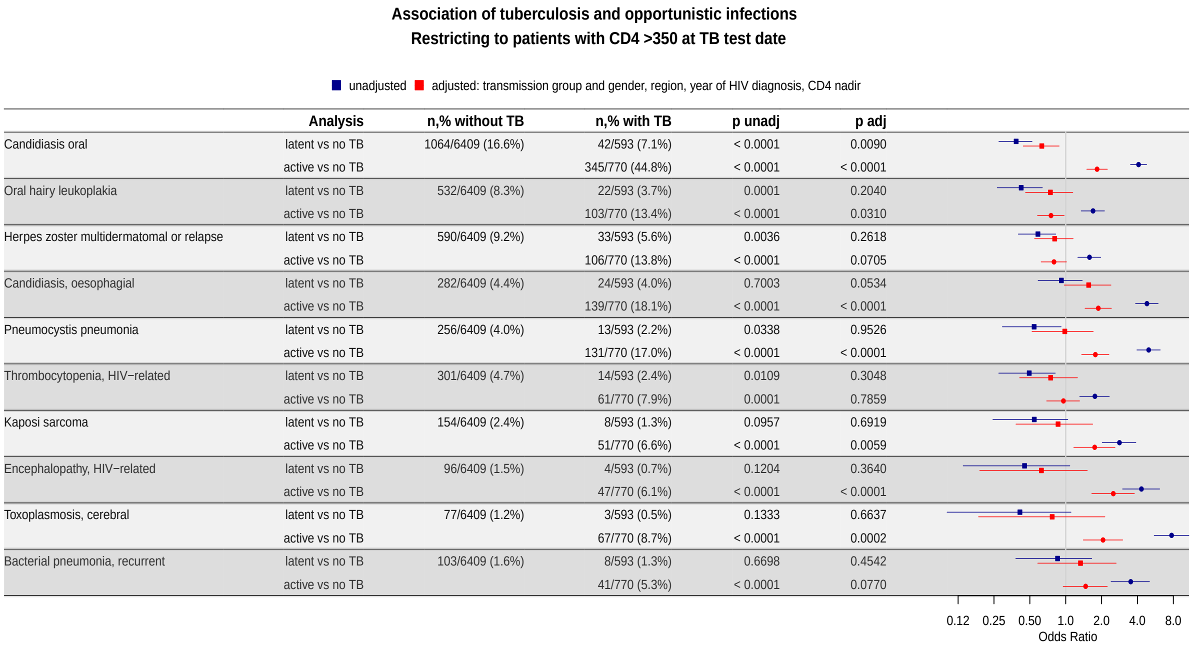

Fig J. Association of the ten most frequent opportunistic infections with tuberculosis (TB) infection: Patients with active MTB infection and latent tuberculosis infection (LTBI) compared to tuberculosis uninfected patients, respectively (active vs no TB, latent vs no TB)

Association of tuberculosis and opportunistic infections
Restricting to patients with CD4 >500 at TB test date

■ unadjusted ■ adjusted: transmission group and gender, region, year of HIV diagnosis, CD4 nadir

	Analysis	n,% without TB	n,% with TB	p unadj	p adj
Candidiasis oral	latent vs no TB	561/3893 (14.4%)	27/412 (6.6%)	< 0.0001	0.0339
	active vs no TB		345/770 (44.8%)	< 0.0001	< 0.0001
Oral hairy leukoplakia	latent vs no TB	284/3893 (7.3%)	13/412 (3.2%)	0.0020	0.1114
	active vs no TB		103/770 (13.4%)	< 0.0001	0.0896
Herpes zoster multidermatomal or relapse	latent vs no TB	335/3893 (8.6%)	18/412 (4.4%)	0.0037	0.0539
	active vs no TB		106/770 (13.8%)	< 0.0001	0.0782
Candidiasis, oesophagial	latent vs no TB	140/3893 (3.6%)	14/412 (3.4%)	0.7967	0.3001
	active vs no TB		139/770 (18.1%)	< 0.0001	< 0.0001
Pneumocystis pneumonia	latent vs no TB	128/3893 (3.3%)	6/412 (1.5%)	0.0456	0.4169
	active vs no TB		131/770 (17.0%)	< 0.0001	< 0.0001
Thrombocytopenia, HIV−related	latent vs no TB	171/3893 (4.4%)	9/412 (2.2%)	0.0355	0.2511
	active vs no TB		61/770 (7.9%)	< 0.0001	0.4200
Kaposi sarcoma	latent vs no TB	90/3893 (2.3%)	7/412 (1.7%)	0.4444	0.9309
	active vs no TB		51/770 (6.6%)	< 0.0001	0.0172
Encephalopathy, HIV−related	latent vs no TB	58/3893 (1.5%)	2/412 (0.5%)	0.1054	0.2311
	active vs no TB		47/770 (6.1%)	< 0.0001	0.0003
Toxoplasmosis, cerebral	latent vs no TB	39/3893 (1.0%)	0/412 (0.0%)		
	active vs no TB		67/770 (8.7%)	< 0.0001	0.0050
Bacterial pneumonia, recurrent	latent vs no TB	58/3893 (1.5%)	5/412 (1.2%)	0.6308	0.7596
	active vs no TB		41/770 (5.3%)	< 0.0001	0.0975

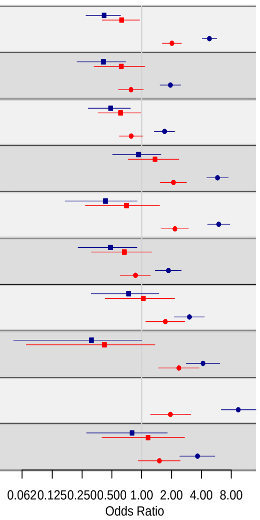

Fig K. Association of the ten most frequent opportunistic infections with tuberculosis (TB) infection: Patients with active MTB infection and latent tuberculosis infection (LTBI) compared to tuberculosis uninfected patients, respectively (active vs no TB, latent vs no TB)

Association of tuberculosis and opportunistic infections
Correction for CD4 count at TB test date

■ unadjusted ■ adjusted: transmission group and gender, region, year of HIV diagnosis, CD4 at TB test date

	Analysis	n,% without TB	n,% with TB	p unadj	p adj
Candidiasis oral	latent vs no TB	3429/12333 (27.8%)	81/840 (9.6%)	< 0.0001	0.0003
	active vs no TB		345/770 (44.8%)	< 0.0001	< 0.0001
Oral hairy leukoplakia	latent vs no TB	1640/12333 (13.3%)	34/840 (4.0%)	< 0.0001	0.0216
	active vs no TB		103/770 (13.4%)	0.9244	0.2604
Herpes zoster multidermatomal or relapse	latent vs no TB	1394/12333 (11.3%)	48/840 (5.7%)	< 0.0001	0.0163
	active vs no TB		106/770 (13.8%)	0.0412	0.0938
Candidiasis, oesophagial	latent vs no TB	1110/12333 (9.0%)	36/840 (4.3%)	< 0.0001	0.9526
	active vs no TB		139/770 (18.1%)	< 0.0001	0.0126
Pneumocystis pneumonia	latent vs no TB	1110/12333 (9.0%)	25/840 (3.0%)	< 0.0001	0.1161
	active vs no TB		131/770 (17.0%)	< 0.0001	0.0696
Thrombocytopenia, HIV−related	latent vs no TB	802/12333 (6.5%)	28/840 (3.3%)	0.0003	0.4934
	active vs no TB		61/770 (7.9%)	0.1313	0.0727
Kaposi sarcoma	latent vs no TB	567/12333 (4.6%)	12/840 (1.4%)	< 0.0001	0.2370
	active vs no TB		51/770 (6.6%)	0.0113	0.4317
Encephalopathy, HIV−related	latent vs no TB	395/12333 (3.2%)	7/840 (0.8%)	0.0003	0.1035
	active vs no TB		47/770 (6.1%)	< 0.0001	0.4310
Toxoplasmosis, cerebral	latent vs no TB	345/12333 (2.8%)	7/840 (0.8%)	0.0012	0.1542
	active vs no TB		67/770 (8.7%)	< 0.0001	0.0273
Bacterial pneumonia, recurrent	latent vs no TB	345/12333 (2.8%)	8/840 (1.0%)	0.0022	0.4601
	active vs no TB		41/770 (5.3%)	< 0.0001	0.0258

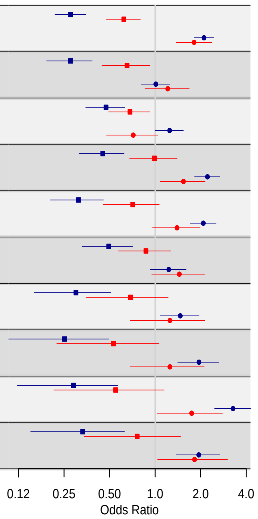

Fig L. Association of the ten most frequent opportunistic infections with tuberculosis (TB) infection: Patients with active MTB infection and latent tuberculosis infection (LTBI) compared to tuberculosis uninfected patients, respectively (active vs no TB, latent vs no TB)

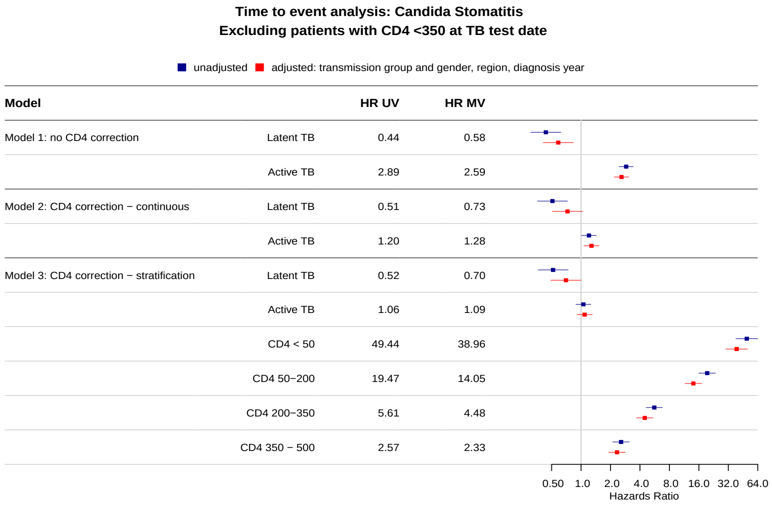

Fig M. Time to event analysis of the occurrence of candida stomatitis: Patients with active MTB infection or latent MTB infection compared to tuberculosis uninfected patients, respectively.

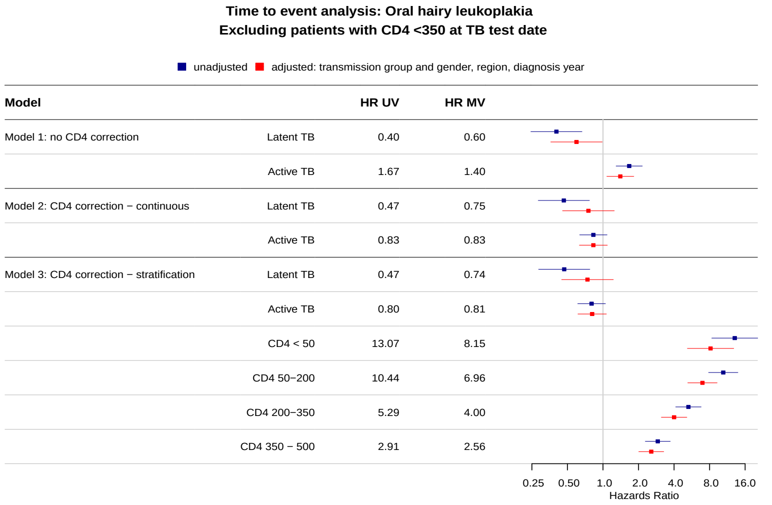

Fig N. Time to event analysis of the occurrence of oral hairy leukoplakia: Patients with active MTB infection or latent MTB infection compared to tuberculosis uninfected patients, respectively.

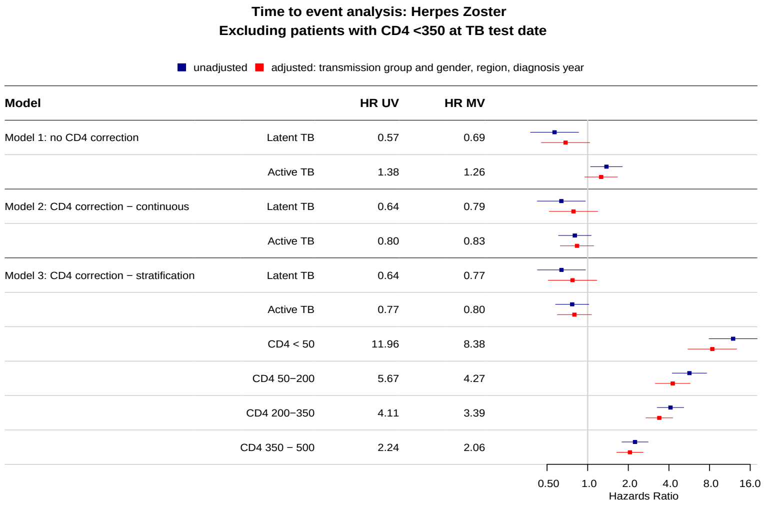

Model		HR UV	HR MV
Model 1: no CD4 correction	Latent TB	0.57	0.69
	Active TB	1.38	1.26
Model 2: CD4 correction − continuous	Latent TB	0.64	0.79
	Active TB	0.80	0.83
Model 3: CD4 correction − stratification	Latent TB	0.64	0.77
	Active TB	0.77	0.80
	CD4 < 50	11.96	8.38
	CD4 50−200	5.67	4.27
	CD4 200−350	4.11	3.39
	CD4 350 − 500	2.24	2.06

Fig O. Time to event analysis of the occurrence of herpes zoster: Patients with active MTB infection or latent MTB infection compared to tuberculosis uninfected patients, respectively.

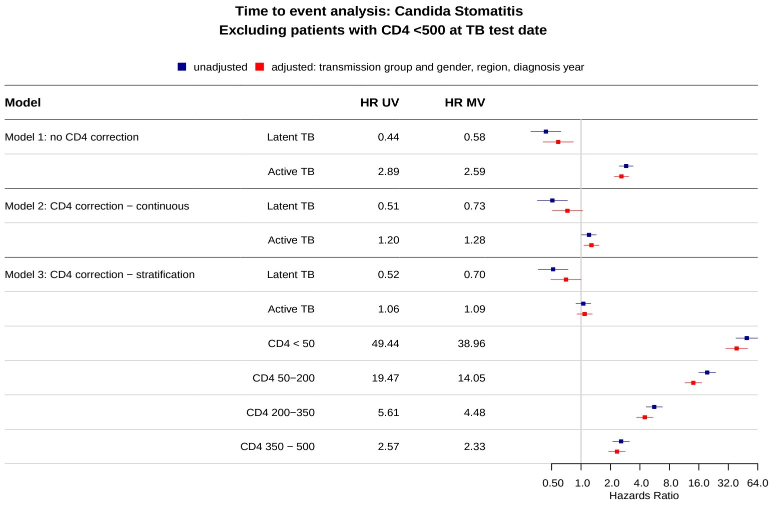

Model		HR UV	HR MV
Model 1: no CD4 correction	Latent TB	0.44	0.58
	Active TB	2.89	2.59
Model 2: CD4 correction − continuous	Latent TB	0.51	0.73
	Active TB	1.20	1.28
Model 3: CD4 correction − stratification	Latent TB	0.52	0.70
	Active TB	1.06	1.09
	CD4 < 50	49.44	38.96
	CD4 50−200	19.47	14.05
	CD4 200−350	5.61	4.48
	CD4 350 − 500	2.57	2.33

Fig P. Time to event analysis of the occurrence of candida stomatitis: Patients with active MTB infection or latent MTB infection compared to tuberculosis uninfected patients, respectively.

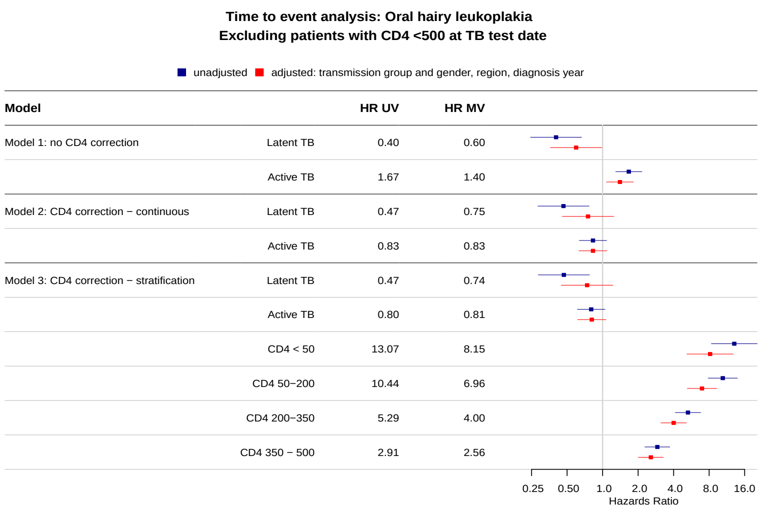

Fig Q. Time to event analysis of the occurrence of oral hairy leukoplakia: Patients with active MTB infection or latent MTB infection compared to tuberculosis uninfected patients, respectively.

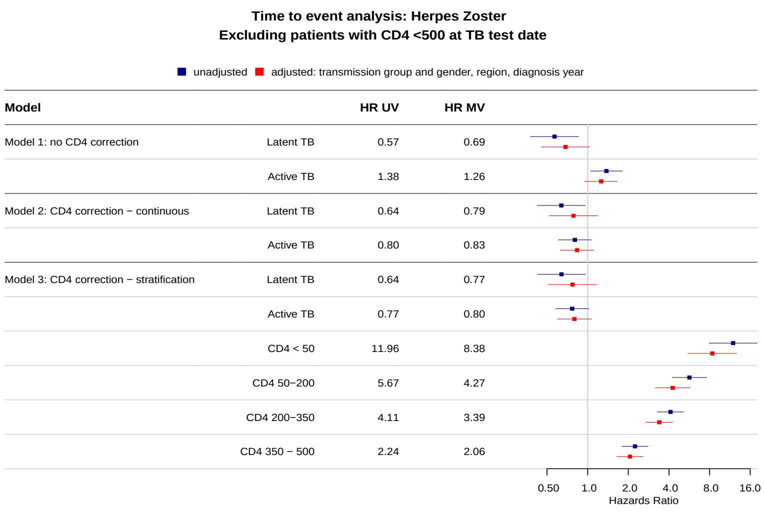

Fig R. Time to event analysis of the occurrence of herpes zoster: Patients with active MTB infection or latent MTB infection compared to tuberculosis uninfected patients, respectively.

2 Timing of the tuberculosis test

2.1 Tuberculosis test within 1 year of SHCS registration

In this subanalysis, we included only patients who had a tuberculosis test or clinically diagnosed active tuberculosis diagnosed within 1 year of the SHCS registration. Of the 9884 patients, 8673 had no tuberculosis test, 673 latent tuberculosis infection and 538 active tuberculosis.

A) HIV set point virus load

	Original results	Tuberculosis test ± 1 year of SHCS registration
Sample size	4516	3229
Overall mean:	4.4	4.4
Mean: No tuberculosis	4.43	4.43
Mean: LTBI	4.11	4.08
Mean: Active MTB infection	4.63	4.9
unadjusted: LTBI	-0.32 [-0.4, -0.24]	-0.34 [-0.43, -0.25]
unadjusted: active MTB infection	0.2 [0.03, 0.37]	0.47 [0.21, 0.74]
adjusted: LTBI	-0.21 [-0.28, -0.13]	-0.23 [-0.32, -0.15]
adjusted: active MTB infection	0.14 [-0.02, 0.31]	0.38 [0.12, 0.63]

B) Opportunistic infections

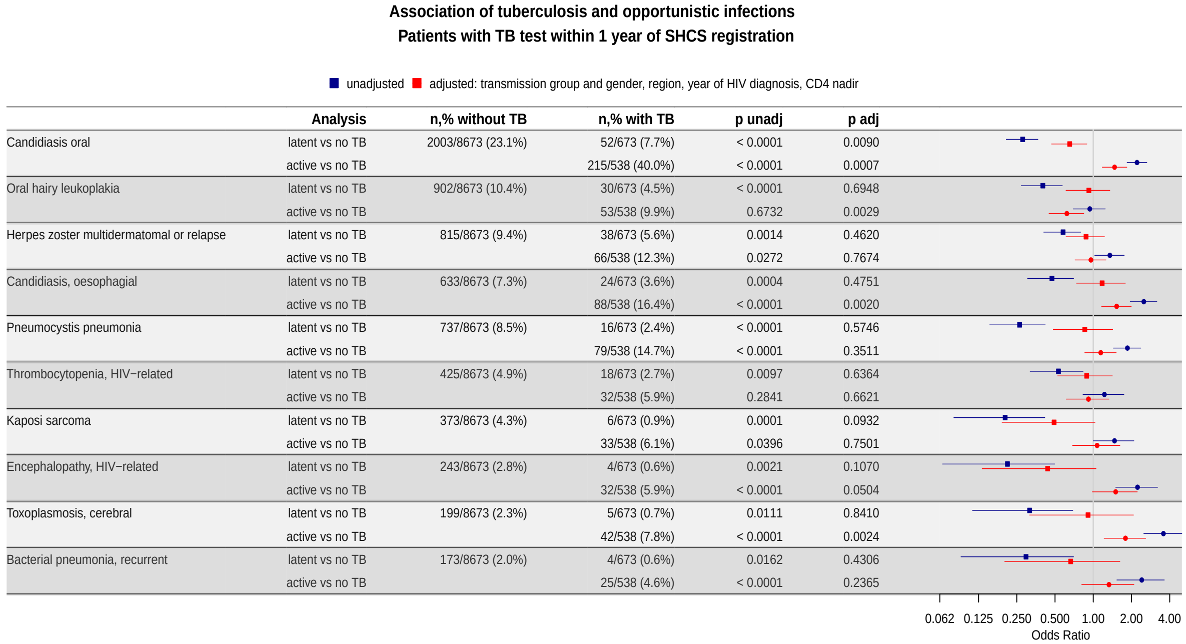

Fig S. Association of the ten most frequent opportunistic infections with tuberculosis (TB) infection: Patients with active MTB infection and latent tuberculosis infection (LTBI) compared to tuberculosis uninfected patients, respectively (active vs no TB, latent vs no TB)

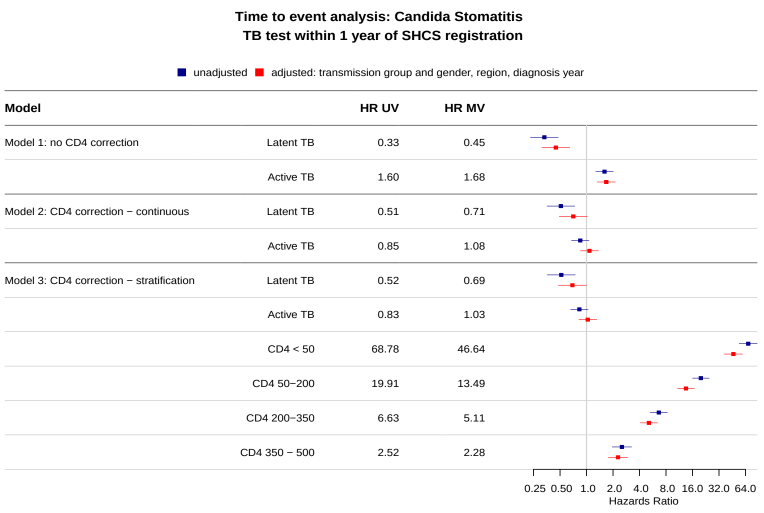

Fig T. Time to event analysis of the occurrence of candida stomatitis: Patients with active MTB infection or latent MTB infection compared to tuberculosis uninfected patients, respectively.

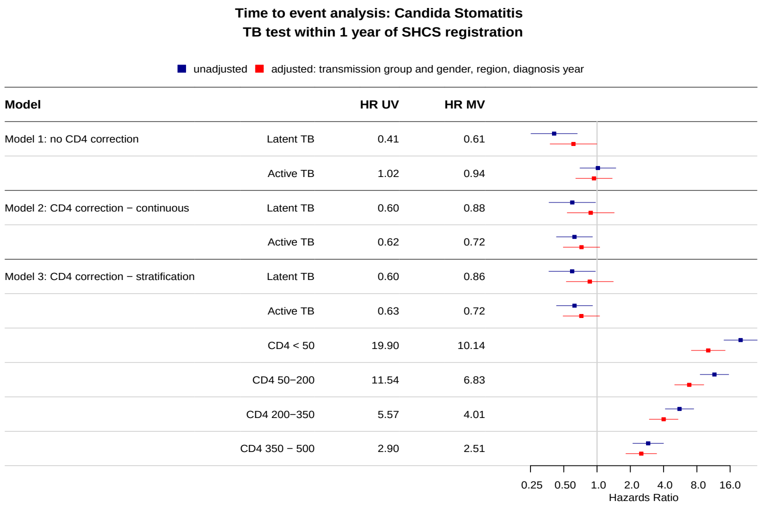

Fig U. Time to event analysis of the occurrence of oral hairy leukoplakia: Patients with active MTB infection or latent MTB infection compared to tuberculosis uninfected patients, respectively.

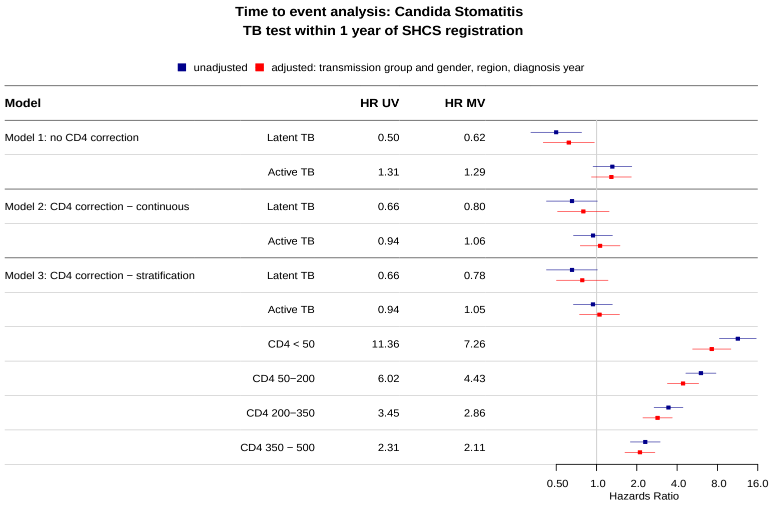

Fig V. Time to event analysis of the occurrence of herpes zoster: Patients with active MTB infection or latent MTB infection compared to tuberculosis uninfected patients, respectively.

2.2 Timing of opportunistic infections

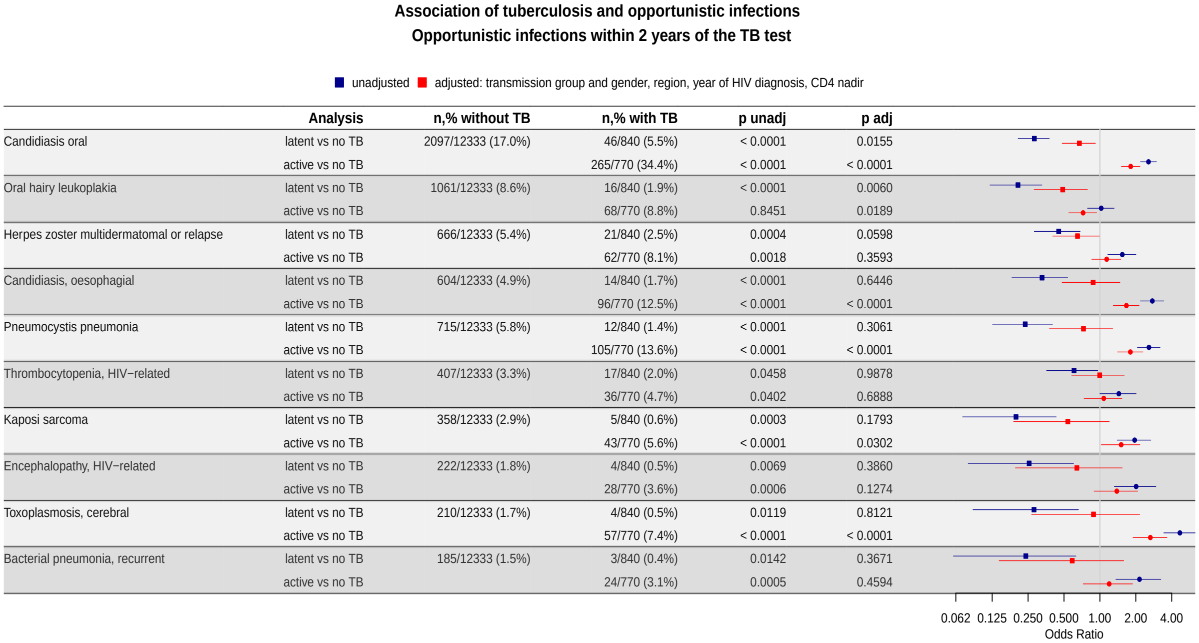

Fig W. Association of the ten most frequent opportunistic infections with tuberculosis (TB) infection: Patients with active MTB infection and latent tuberculosis infection (LTBI) compared to tuberculosis uninfected patients, respectively (active vs no TB, latent vs no TB)

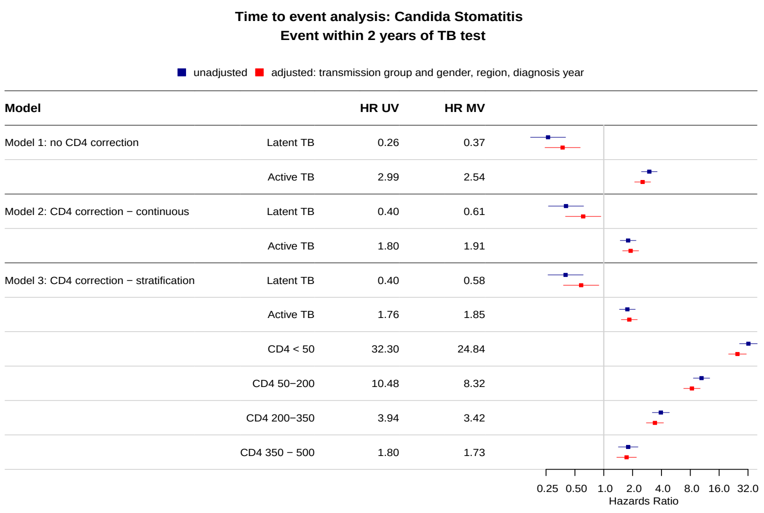

Fig X. Time to event analysis of the occurrence of candida stomatitis: Patients with active MTB infection or latent MTB infection compared to tuberculosis uninfected patients, respectively.

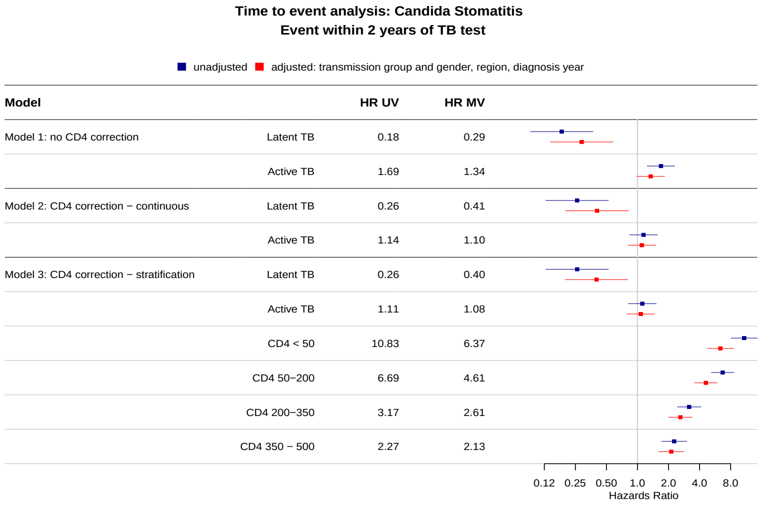

Fig Y. Time to event analysis of the occurrence of oral hairy leukoplakia: Patients with active MTB infection or latent MTB infection compared to tuberculosis uninfected patients, respectively.

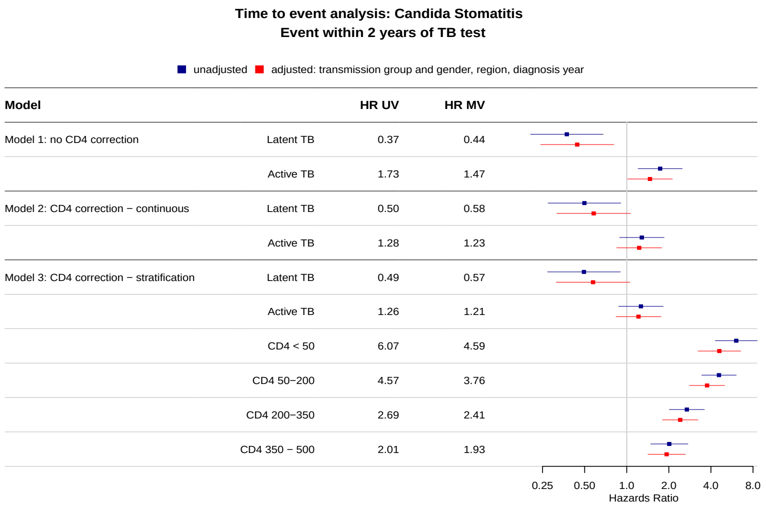

Fig Z. Time to event analysis of the occurrence of herpes zoster: Patients with active MTB infection or latent MTB infection compared to tuberculosis uninfected patients, respectively.

3 Antiretroviral treatment

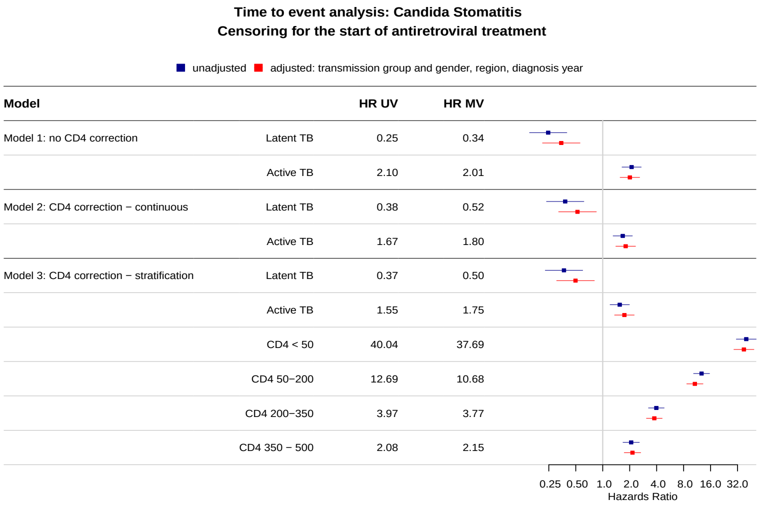

Fig AA. Time to event analysis of the occurrence of candida stomatitis: Patients with active MTB infection or latent MTB infection compared to tuberculosis uninfected patients, respectively.

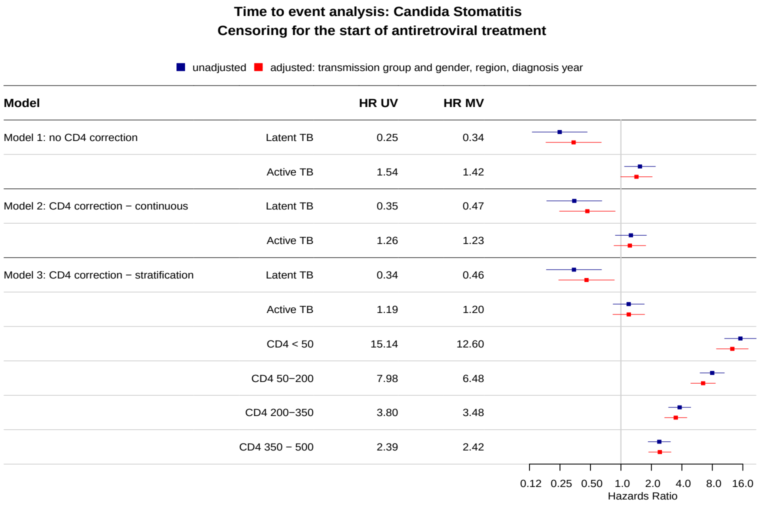

Fig AB. Time to event analysis of the occurrence of oral hairy leukoplakia: Patients with active MTB infection or latent MTB infection compared to tuberculosis uninfected patients, respectively.

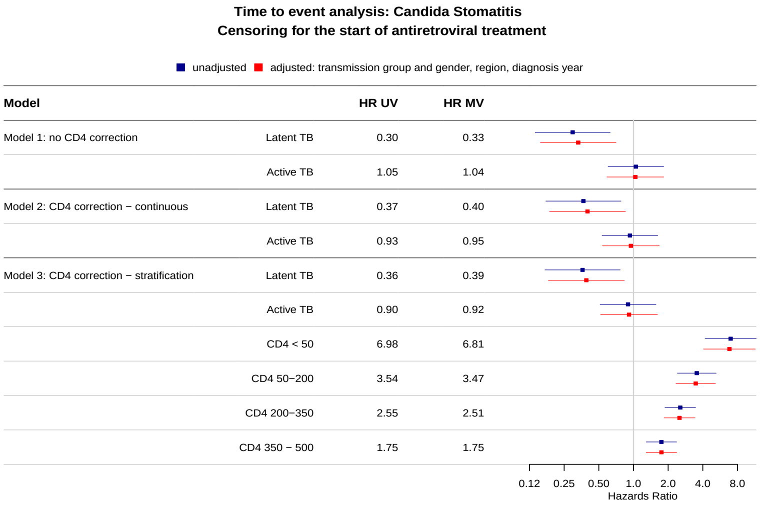

Fig AC. Time to event analysis of the occurrence of herpes zoster: Patients with active MTB infection or latent MTB infection compared to tuberculosis uninfected patients, respectively.

4 Prophylactic tuberculosis treatment

Of the 840 patients with LTBI, 406 (48.3%) obtained prophylactic treatment, i.e., Rifampicin or Isozianid. Excluding these 406 patients from the group of LTBI patients yield the following results.

A) HIV set point virus load

	Original results	Excluding patients with prophylactic treatment
Sample size	4516	4335
Overall mean:	4.4	4.42
Mean: No tuberculosis	4.43	4.43
Mean: LTBI	4.11	4.12
Mean: Active MTB infection	4.63	4.63
unadjusted: LTBI	-0.32 [-0.4, -0.24]	-0.3 [-0.41, -0.2]
unadjusted: active MTB infection	0.2 [0.03, 0.37]	0.2 [0.03, 0.37]
adjusted: LTBI	-0.21 [-0.28, -0.13]	-0.21 [-0.31, -0.11]
adjusted: active MTB infection	0.14 [-0.02, 0.31]	0.14 [-0.02, 0.31]

B) Opportunistic infections

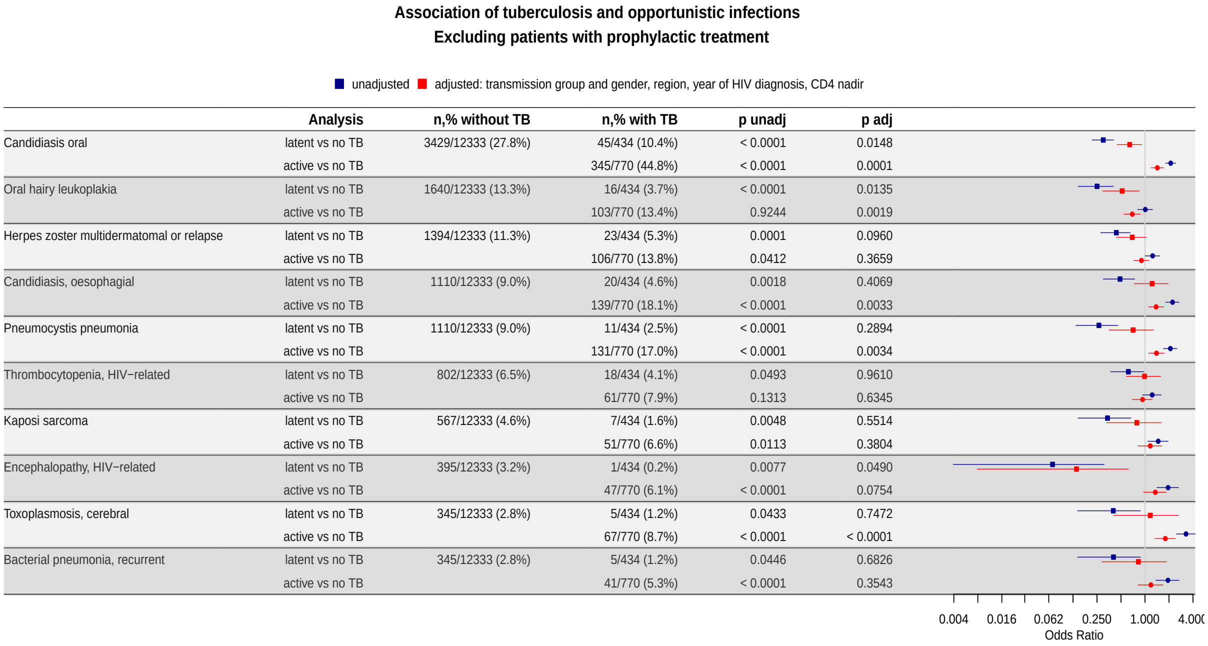

Fig AD. Association of the ten most frequent opportunistic infections with tuberculosis (TB) infection: Patients with active MTB infection and latent tuberculosis infection (LTBI) compared to tuberculosis uninfected patients, respectively (active vs no TB, latent vs no TB)

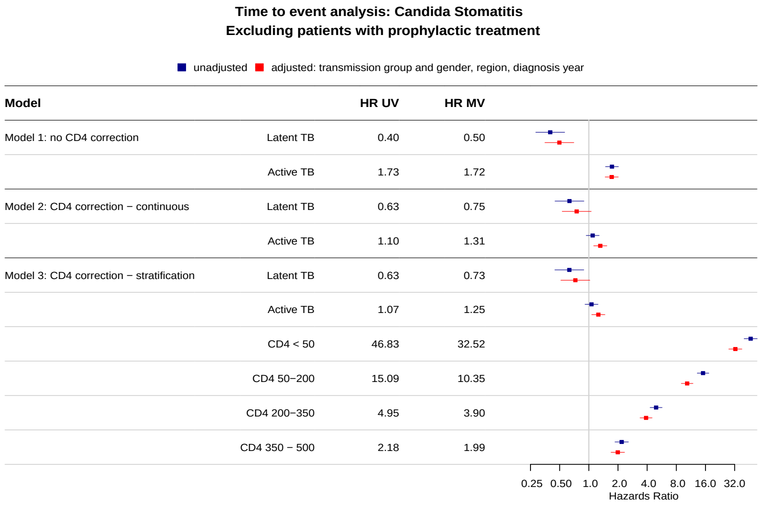

Fig AE. Time to event analysis of the occurrence of candida stomatitis: Patients with active MTB infection or latent MTB infection compared to tuberculosis uninfected patients, respectively.

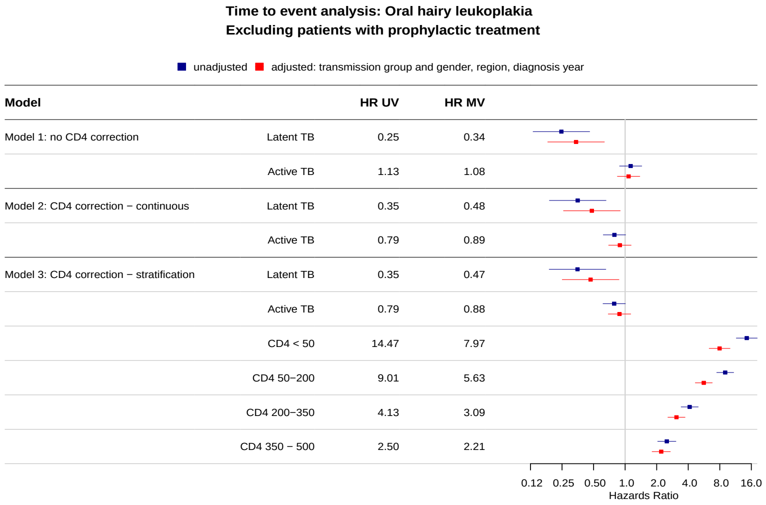

Fig AF. Time to event analysis of the occurrence of oral hairy leukoplakia: Patients with active MTB infection or latent MTB infection compared to tuberculosis uninfected patients, respectively.

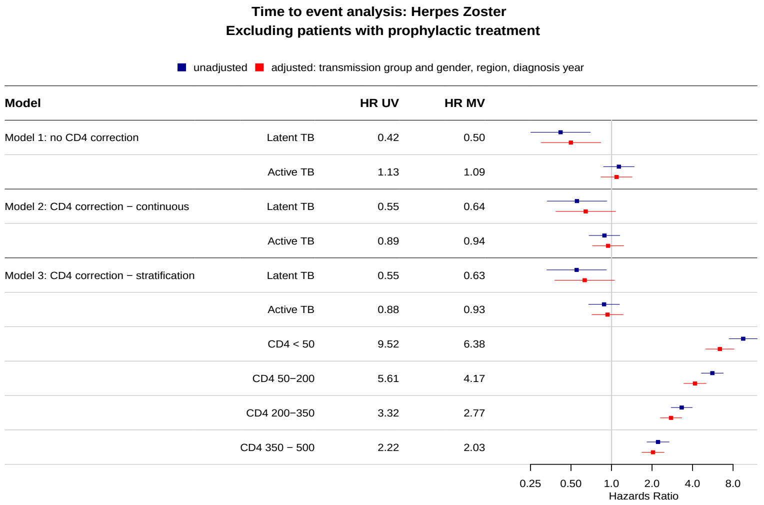

Fig AG. Time to event analysis of the occurrence of herpes zoster: Patients with active MTB infection or latent MTB infection compared to tuberculosis uninfected patients, respectively.

5 Ethnicity, region and HIV subtype

To better understand the impact of ethnicity and region on our results, we performed the following three sensitivity analyses:

1. Restricting to patients infected with HIV subtype B
2. Instead of correcting for the region of the patients, we correct for the ethnicity, grouped by white, black and other (including Asian, Hispano-American, unknown and other ethnicities).
3. Restricting the analyses to patients of white ethnicity
4. Restricting the analyses to patients from Western Europe

A) HIV set point virus load

	Original results	Restriction to subtype B
Sample size	4516	2846
Overall mean:	4.4	4.45
Mean: No tuberculosis	4.43	4.47
Mean: LTBI	4.11	4.12
Mean: Active MTB infection	4.63	4.51
unadjusted: LTBI	-0.32 [-0.4, -0.24]	-0.36 [-0.46, -0.25]
unadjusted: active MTB infection	0.2 [0.03, 0.37]	0.03 [-0.2, 0.27]
adjusted: LTBI	-0.21 [-0.28, -0.13]	-0.26 [-0.36, -0.17]
adjusted: active MTB infection	0.14 [-0.02, 0.31]	-0.04 [-0.26, 0.19]

	Original	Ethnicity group	Only white patients	Only patients from Western Europe
Sample size	4516	4516	3617	3237
Overall mean:	4.4	4.4	4.45	4.44
Mean: No tuberculosis	4.43	4.43	4.47	4.46
Mean: LTBI	4.11	4.11	4.14	4.15
Mean: Active MTB infection	4.63	4.63	4.56	4.7
unadjusted: LTBI	-0.32 [-0.4, -0.24]	-0.32 [-0.4, -0.24]	-0.33 [-0.43, -0.23]	-0.31 [-0.41, -0.2]
unadjusted: active MTB infection	0.2 [0.03, 0.37]	0.2 [0.03, 0.37]	0.09 [-0.15, 0.32]	0.24 [-0.01, 0.49]
adjusted: LTBI	-0.21 [-0.28, -0.13]	-0.2 [-0.28, -0.12]	-0.26 [-0.35, -0.16]	-0.22 [-0.32, -0.11]
adjusted: active MTB infection	0.14 [-0.02, 0.31]	0.16 [-0.01, 0.32]	-0.02 [-0.24, 0.21]	0.14 [-0.09, 0.38]

B) Opportunistic infections

Association of tuberculosis and opportunistic infections
Restriction to patients with HIV subtype B

■ unadjusted ■ adjusted: transmission group and gender, year of HIV diagnosis, CD4 nadir

	Analysis	n,% without TB	n,% with TB	p unadj	p adj
Candidiasis oral	latent vs no TB	2018/6079 (33.2%)	48/314 (15.3%)	< 0.0001	0.0280
	active vs no TB		100/193 (51.8%)	< 0.0001	0.4998
Oral hairy leukoplakia	latent vs no TB	1040/6079 (17.1%)	17/314 (5.4%)	< 0.0001	0.0034
	active vs no TB		38/193 (19.7%)	0.3403	0.0893
Herpes zoster multidermatomal or relapse	latent vs no TB	881/6079 (14.5%)	19/314 (6.1%)	< 0.0001	0.0300
	active vs no TB		38/193 (19.7%)	0.0464	0.8404
Candidiasis, oesophagial	latent vs no TB	608/6079 (10.0%)	19/314 (6.1%)	0.0224	0.4350
	active vs no TB		33/193 (17.1%)	0.0017	0.9719
Pneumocystis pneumonia	latent vs no TB	541/6079 (8.9%)	16/314 (5.1%)	0.0225	0.1648
	active vs no TB		28/193 (14.5%)	0.0079	0.8094
Thrombocytopenia, HIV−related	latent vs no TB	559/6079 (9.2%)	18/314 (5.7%)	0.0395	0.7204
	active vs no TB		31/193 (16.1%)	0.0015	0.5248
Kaposi sarcoma	latent vs no TB	316/6079 (5.2%)	7/314 (2.2%)	0.0238	0.4738
	active vs no TB		8/193 (4.1%)	0.5221	0.4906
Encephalopathy, HIV−related	latent vs no TB	182/6079 (3.0%)	3/314 (1.0%)	0.0437	0.3022
	active vs no TB		8/193 (4.1%)	0.3849	0.7197
Toxoplasmosis, cerebral	latent vs no TB	146/6079 (2.4%)	3/314 (1.0%)	0.1101	0.9920
	active vs no TB		16/193 (8.3%)	< 0.0001	0.0189
Bacterial pneumonia, recurrent	latent vs no TB	182/6079 (3.0%)	6/314 (1.9%)	0.2842	0.9723
	active vs no TB		15/193 (7.8%)	0.0003	0.1017

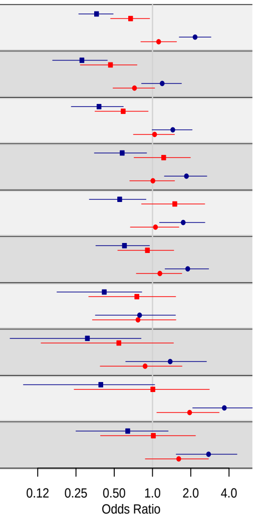

Fig AH. Association of the ten most frequent opportunistic infections with tuberculosis (TB) infection: Patients with active MTB infection and latent tuberculosis infection (LTBI) compared to tuberculosis uninfected patients, respectively (active vs no TB, latent vs no TB)

Association of tuberculosis and opportunistic infections
White, black or other ethnicity

■ unadjusted ■ adjusted: transmission group and gender, ethnicity (white, black, other), year of HIV diagnosis, CD4 nadir

	Analysis	n,% without TB	n,% with TB	p unadj	p adj
Candidiasis oral	latent vs no TB	3429/12333 (27.8%)	81/840 (9.6%)	< 0.0001	0.0033
	active vs no TB		345/770 (44.8%)	< 0.0001	0.0017
Oral hairy leukoplakia	latent vs no TB	1640/12333 (13.3%)	34/840 (4.0%)	< 0.0001	0.0408
	active vs no TB		103/770 (13.4%)	0.9244	0.0003
Herpes zoster multidermatomal or relapse	latent vs no TB	1394/12333 (11.3%)	48/840 (5.7%)	< 0.0001	0.1071
	active vs no TB		106/770 (13.8%)	0.0412	0.4904
Candidiasis, oesophagial	latent vs no TB	1110/12333 (9.0%)	36/840 (4.3%)	< 0.0001	0.2976
	active vs no TB		139/770 (18.1%)	< 0.0001	0.0145
Pneumocystis pneumonia	latent vs no TB	1110/12333 (9.0%)	25/840 (3.0%)	< 0.0001	0.6722
	active vs no TB		131/770 (17.0%)	< 0.0001	0.0039
Thrombocytopenia, HIV−related	latent vs no TB	802/12333 (6.5%)	28/840 (3.3%)	0.0003	0.5347
	active vs no TB		61/770 (7.9%)	0.1313	0.9620
Kaposi sarcoma	latent vs no TB	567/12333 (4.6%)	12/840 (1.4%)	< 0.0001	0.3485
	active vs no TB		51/770 (6.6%)	0.0113	0.6417
Encephalopathy, HIV−related	latent vs no TB	395/12333 (3.2%)	7/840 (0.8%)	0.0003	0.1350
	active vs no TB		47/770 (6.1%)	< 0.0001	0.4864
Toxoplasmosis, cerebral	latent vs no TB	345/12333 (2.8%)	7/840 (0.8%)	0.0012	0.6333
	active vs no TB		67/770 (8.7%)	< 0.0001	0.0009
Bacterial pneumonia, recurrent	latent vs no TB	345/12333 (2.8%)	8/840 (1.0%)	0.0022	0.5271
	active vs no TB		41/770 (5.3%)	< 0.0001	0.8736

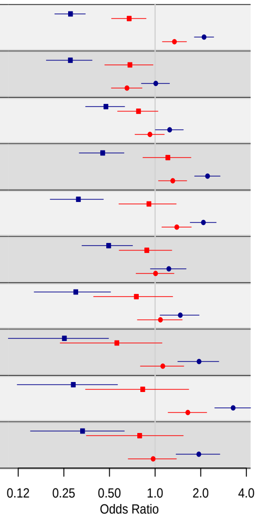

Fig AI. Association of the ten most frequent opportunistic infections with tuberculosis (TB) infection: Patients with active MTB infection and latent tuberculosis infection (LTBI) compared to tuberculosis uninfected patients, respectively (active vs no TB, latent vs no TB)

Association of tuberculosis and opportunistic infections
Restriction to patients of white ethnicity

■ unadjusted ■ adjusted: transmission group and gender, year of HIV diagnosis, CD4 nadir

	Analysis	n,% without TB	n,% with TB	p unadj	p adj
Candidiasis oral	latent vs no TB	2650/9364 (28.3%)	64/459 (13.9%)	< 0.0001	0.0520
	active vs no TB		165/299 (55.2%)	< 0.0001	0.0039
Oral hairy leukoplakia	latent vs no TB	1274/9364 (13.6%)	29/459 (6.3%)	< 0.0001	0.1691
	active vs no TB		57/299 (19.1%)	0.0069	0.1050
Herpes zoster multidermatomal or relapse	latent vs no TB	1067/9364 (11.4%)	27/459 (5.9%)	0.0003	0.1017
	active vs no TB		44/299 (14.7%)	0.0784	0.4728
Candidiasis, oesophagial	latent vs no TB	833/9364 (8.9%)	27/459 (5.9%)	0.0265	0.2183
	active vs no TB		62/299 (20.7%)	< 0.0001	0.1296
Pneumocystis pneumonia	latent vs no TB	852/9364 (9.1%)	20/459 (4.4%)	0.0006	0.9094
	active vs no TB		73/299 (24.4%)	< 0.0001	0.0004
Thrombocytopenia, HIV−related	latent vs no TB	693/9364 (7.4%)	21/459 (4.6%)	0.0230	0.4374
	active vs no TB		31/299 (10.4%)	0.0596	0.2319
Kaposi sarcoma	latent vs no TB	440/9364 (4.7%)	9/459 (2.0%)	0.0078	0.4953
	active vs no TB		21/299 (7.0%)	0.0669	0.9833
Encephalopathy, HIV−related	latent vs no TB	262/9364 (2.8%)	7/459 (1.5%)	0.1060	0.7684
	active vs no TB		14/299 (4.7%)	0.0588	0.9913
Toxoplasmosis, cerebral	latent vs no TB	225/9364 (2.4%)	4/459 (0.9%)	0.0450	0.7350
	active vs no TB		24/299 (8.0%)	< 0.0001	0.0204
Bacterial pneumonia, recurrent	latent vs no TB	215/9364 (2.3%)	6/459 (1.3%)	0.1548	0.8244
	active vs no TB		16/299 (5.4%)	0.0012	0.4165

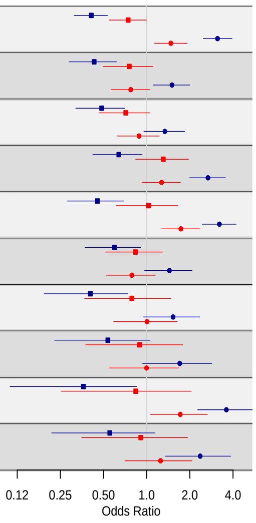

Fig AJ. Association of the ten most frequent opportunistic infections with tuberculosis (TB) infection: Patients with active MTB infection and latent tuberculosis infection (LTBI) compared to tuberculosis uninfected patients, respectively (active vs no TB, latent vs no TB)

Association of tuberculosis and opportunistic infections
Restriction to patients from Western Europe

■ unadjusted ■ adjusted: transmission group and gender, year of HIV diagnosis, CD4 nadir

	Analysis	n,% without TB	n,% with TB	p unadj	p adj
Candidiasis oral	latent vs no TB	2781/8718 (31.9%)	53/374 (14.2%)	< 0.0001	0.0100
	active vs no TB		228/366 (62.3%)	< 0.0001	0.0005
Oral hairy leukoplakia	latent vs no TB	1369/8718 (15.7%)	23/374 (6.1%)	< 0.0001	0.0459
	active vs no TB		75/366 (20.5%)	0.0142	0.0074
Herpes zoster multidermatomal or relapse	latent vs no TB	1055/8718 (12.1%)	22/374 (5.9%)	0.0004	0.0761
	active vs no TB		61/366 (16.7%)	0.0102	0.5747
Candidiasis, oesophagial	latent vs no TB	898/8718 (10.3%)	23/374 (6.1%)	0.0106	0.3810
	active vs no TB		88/366 (24.0%)	< 0.0001	0.0209
Pneumocystis pneumonia	latent vs no TB	863/8718 (9.9%)	17/374 (4.5%)	0.0009	0.6786
	active vs no TB		94/366 (25.7%)	< 0.0001	< 0.0001
Thrombocytopenia, HIV−related	latent vs no TB	663/8718 (7.6%)	19/374 (5.1%)	0.0727	0.6783
	active vs no TB		44/366 (12.0%)	0.0022	0.6498
Kaposi sarcoma	latent vs no TB	462/8718 (5.3%)	7/374 (1.9%)	0.0052	0.4446
	active vs no TB		30/366 (8.2%)	0.0169	0.5492
Encephalopathy, HIV−related	latent vs no TB	331/8718 (3.8%)	6/374 (1.6%)	0.0308	0.4758
	active vs no TB		33/366 (9.0%)	< 0.0001	0.0851
Toxoplasmosis, cerebral	latent vs no TB	262/8718 (3.0%)	4/374 (1.1%)	0.0356	0.8340
	active vs no TB		39/366 (10.7%)	< 0.0001	0.0005
Bacterial pneumonia, recurrent	latent vs no TB	288/8718 (3.3%)	4/374 (1.1%)	0.0226	0.2637
	active vs no TB		25/366 (6.8%)	0.0004	0.8251

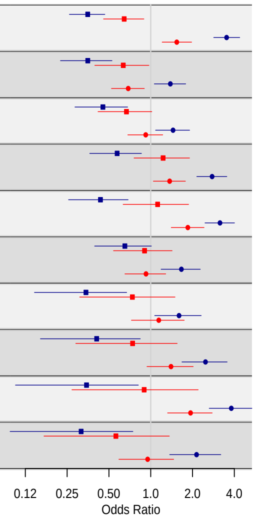

Fig AK. Association of the ten most frequent opportunistic infections with tuberculosis (TB) infection: Patients with active MTB infection and latent tuberculosis infection (LTBI) compared to tuberculosis uninfected patients, respectively (active vs no TB, latent vs no TB)

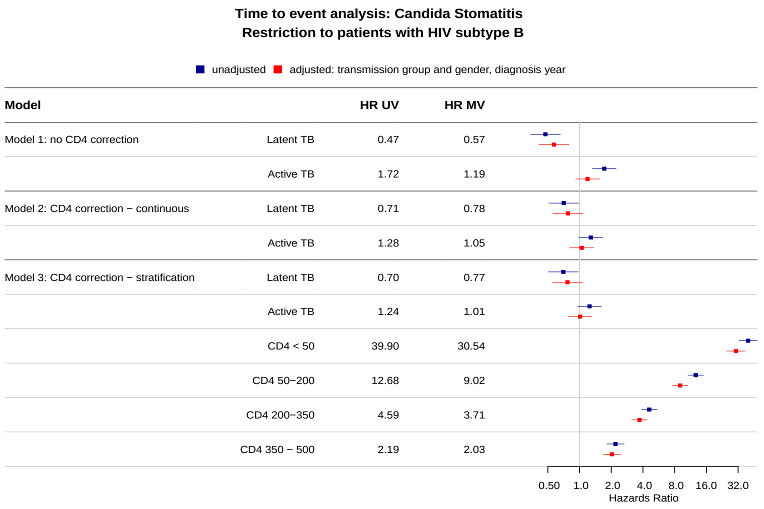

Fig AL. Time to event analysis of the occurrence of candida stomatitis: Patients with active MTB infection or latent MTB infection compared to tuberculosis uninfected patients, respectively.

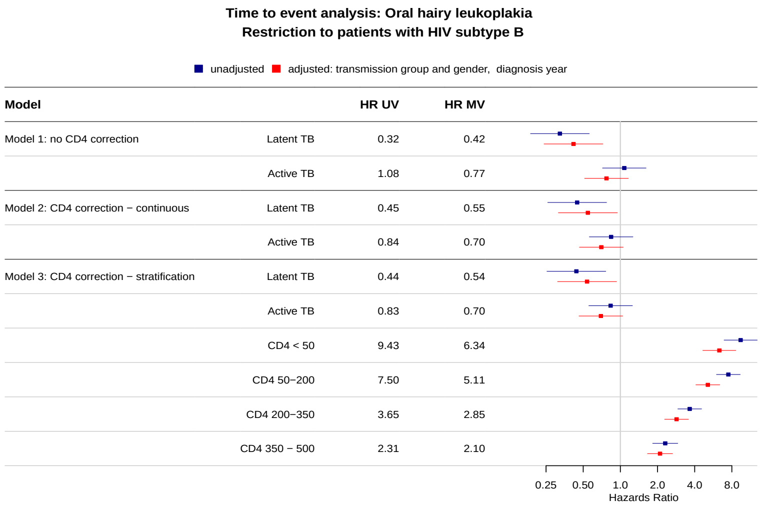

Fig AM. Time to event analysis of the occurrence of oral hairy leukoplakia: Patients with active MTB infection or latent MTB infection compared to tuberculosis uninfected patients, respectively.

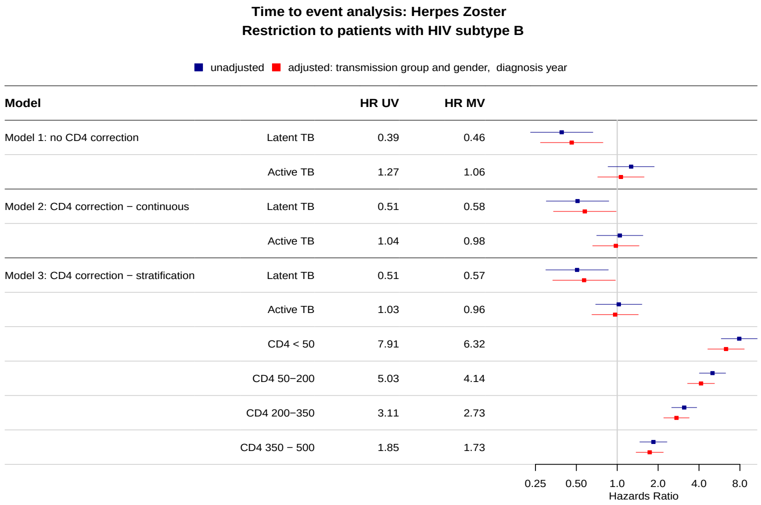

Fig AN. Time to event analysis of the occurrence of herpes zoster: Patients with active MTB infection or latent MTB infection compared to tuberculosis uninfected patients, respectively.

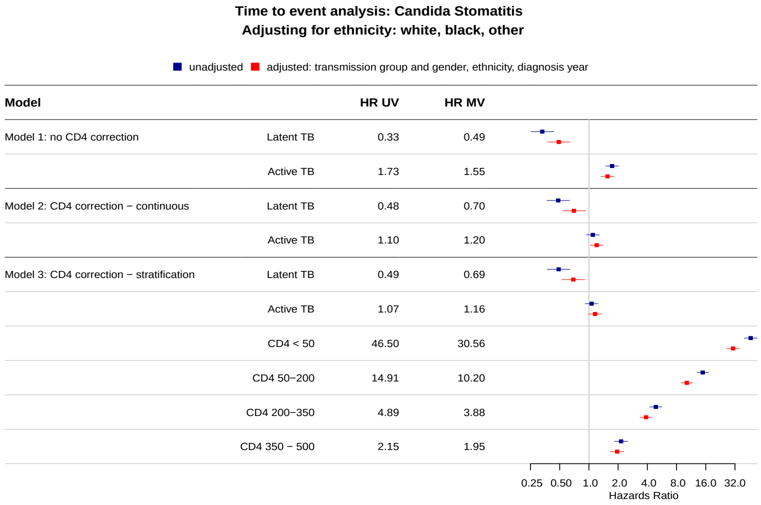

Fig AO. Time to event analysis of the occurrence of candida stomatitis: Patients with active MTB infection or latent MTB infection compared to tuberculosis uninfected patients, respectively.

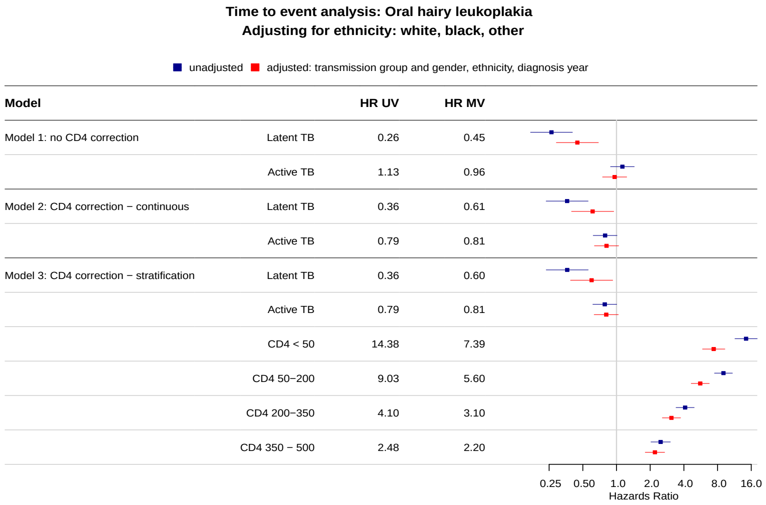

Fig AP. Time to event analysis of the occurrence of oral hairy leukoplakia: Patients with active MTB infection or latent MTB infection compared to tuberculosis uninfected patients, respectively.

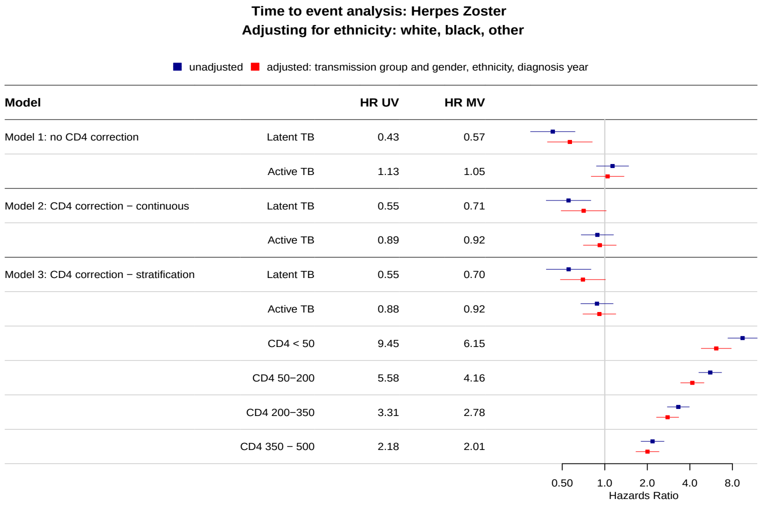

Fig AQ. Time to event analysis of the occurrence of herpes zoster: Patients with active MTB infection or latent MTB infection compared to tuberculosis uninfected patients, respectively.

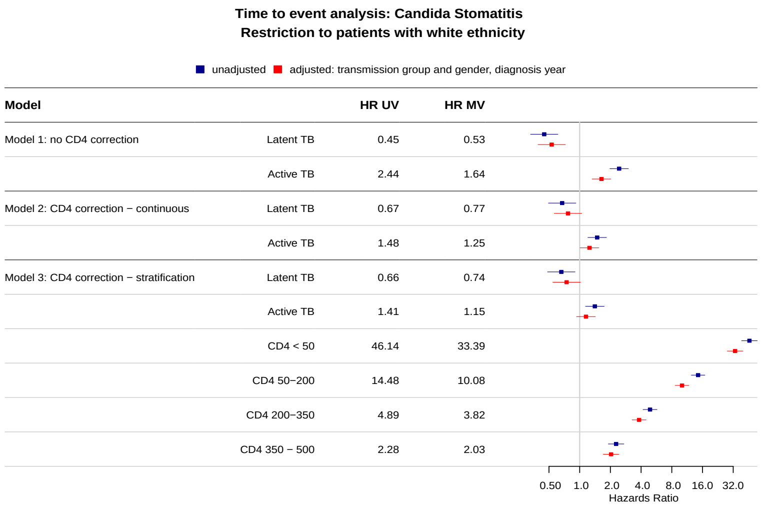

Fig AR. Time to event analysis of the occurrence of candida stomatitis: Patients with active MTB infection or latent MTB infection compared to tuberculosis uninfected patients, respectively.

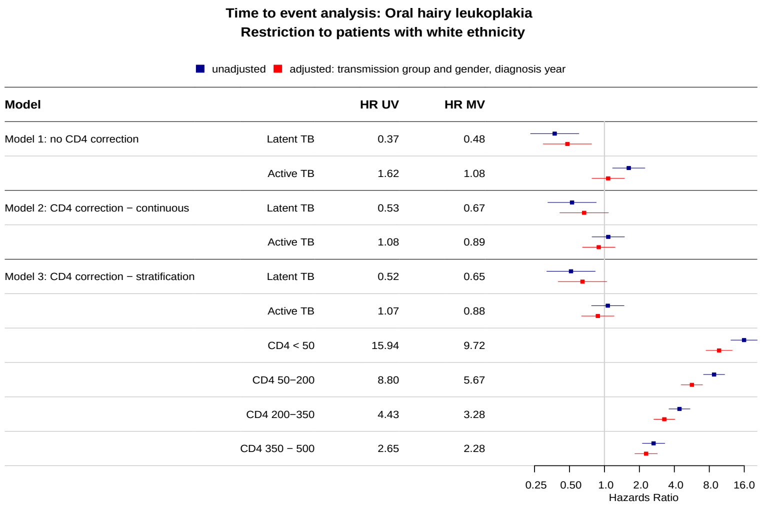

Fig AS. Time to event analysis of the occurrence of oral hairy leukoplakia: Patients with active MTB infection or latent MTB infection compared to tuberculosis uninfected patients, respectively.

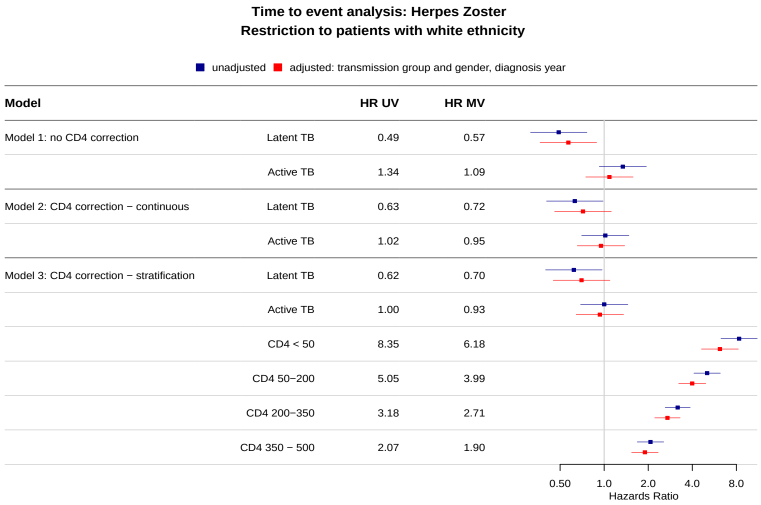

Fig AT. Time to event analysis of the occurrence of herpes zoster: Patients with active MTB infection or latent MTB infection compared to tuberculosis uninfected patients, respectively.

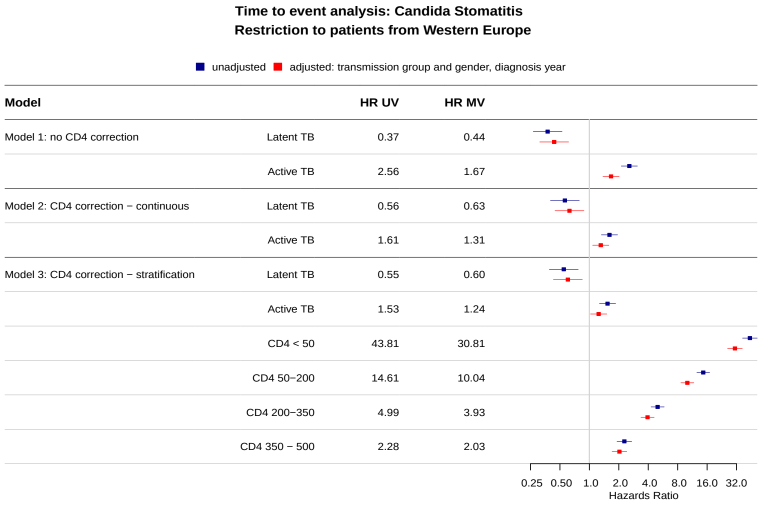

Fig AU. Time to event analysis of the occurrence of candida stomatitis: Patients with active MTB infection or latent MTB infection compared to tuberculosis uninfected patients, respectively.

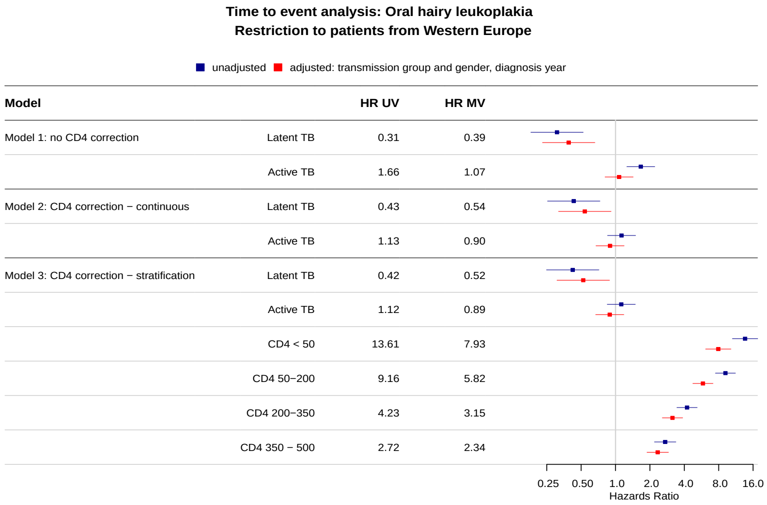

Fig AV. Time to event analysis of the occurrence of oral hairy leukoplakia: Patients with active MTB infection or latent MTB infection compared to tuberculosis uninfected patients, respectively.

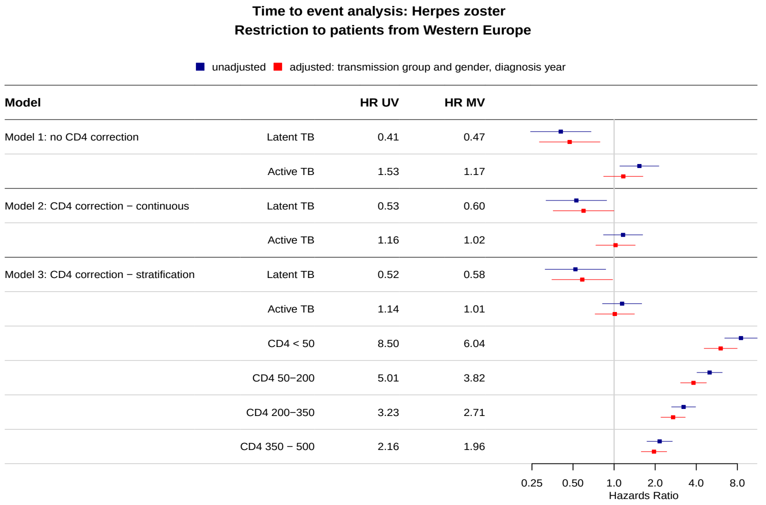

Fig AW. Time to event analysis of the occurrence of herpes zoster: Patients with active MTB infection or latent MTB infection compared to tuberculosis uninfected patients, respectively.

6 Pooling latent and active tuberculosis

A) HIV set point virus load

Of the 4516 patients included in this analysis, 4069 had no tuberculosis infection, 447 were TB infected (latent or active). The overall log mean HIV set point virus load was 4.40 (standard deviation (sd) = 0.75), 4.43 (sd = 0.74) for patients without tuberculosis, 4.19 (sd = 0.75) for patients with tuberculosis. In the unadjusted linear regression model, tuberculosis infection was associated with a 0.23 (confidence interval (CI) = [0.16, 0.31], $p < 0.0001$) decrease in HIV set point virus load, and with 0.15 (CI = [0.08, 0.22], $p < 0.0001$) in the adjusted model.

B) Opportunistic infections

Association of tuberculosis and opportunistic infections
Pooling latent and active tuberculosis

■ unadjusted ■ adjusted: transmission group and gender, region, year of HIV diagnosis, CD4 nadir

	% without TB	% with TB	p unadj	p adj
Candidiasis oral	3429/12333 (27.8%)	427/1610 (26.5%)	0.2430	0.1797
Oral hairy leukoplakia	1640/12333 (13.3%)	137/1610 (8.5%)	< 0.0001	0.0002
Herpes zoster multidermatomal or relapse	1394/12333 (11.3%)	155/1610 (9.6%)	0.0331	0.0893
Candidiasis, oesophagial	1110/12333 (9.0%)	175/1610 (10.9%)	0.0169	0.0025
Pneumocystis pneumonia	1110/12333 (9.0%)	156/1610 (9.7%)	0.3371	0.0184
Thrombocytopenia, HIV−related	802/12333 (6.5%)	89/1610 (5.5%)	0.1242	0.4869
Kaposi sarcoma	567/12333 (4.6%)	63/1610 (3.9%)	0.2042	0.8042
Encephalopathy, HIV−related	395/12333 (3.2%)	55/1610 (3.4%)	0.7868	0.4125
Toxoplasmosis, cerebral	345/12333 (2.8%)	74/1610 (4.6%)	0.0001	0.0008
Bacterial pneumonia, recurrent	345/12333 (2.8%)	48/1610 (3.0%)	0.6016	0.5892

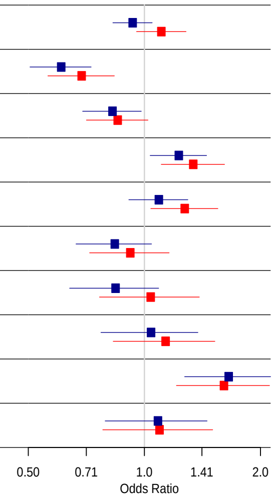

Fig AX. Association of the ten most frequent opportunistic infections with tuberculosis (TB) infection: Patients with tuberculosis infection (latent or active) compared to tuberculosis uninfected patients.

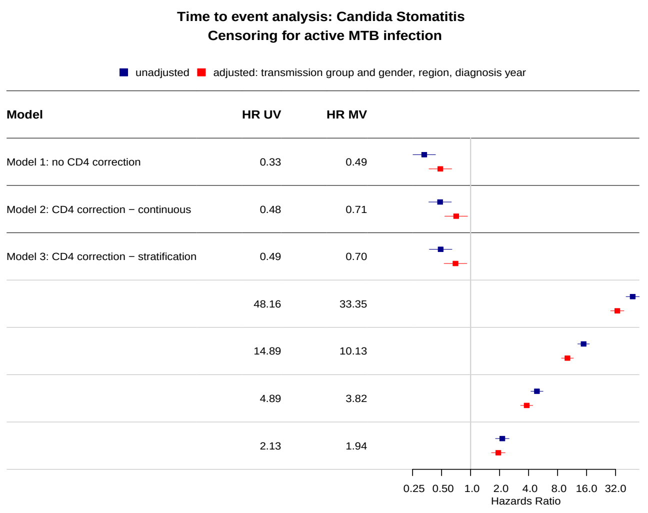

Fig AY. Time to event analysis of the occurrence of candida stomatitis: Patients with tuberculosis infection compared to tuberculosis uninfected patients.

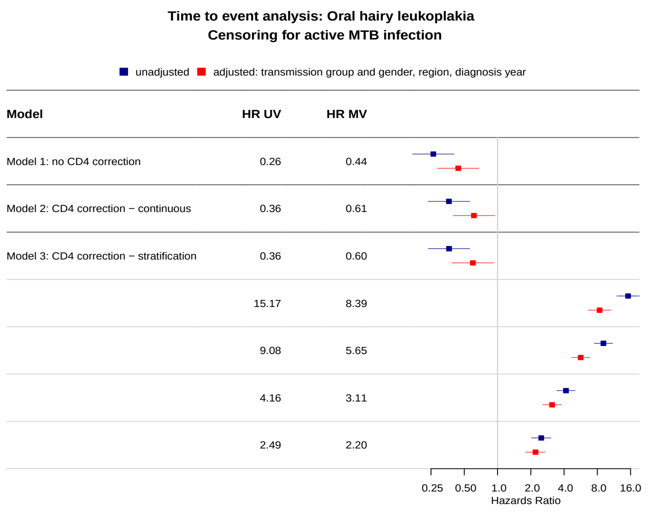

Fig AZ. Time to event analysis of the occurrence of oral hairy leukoplakia: Patients with tuberculosis infection compared to tuberculosis uninfected patients.

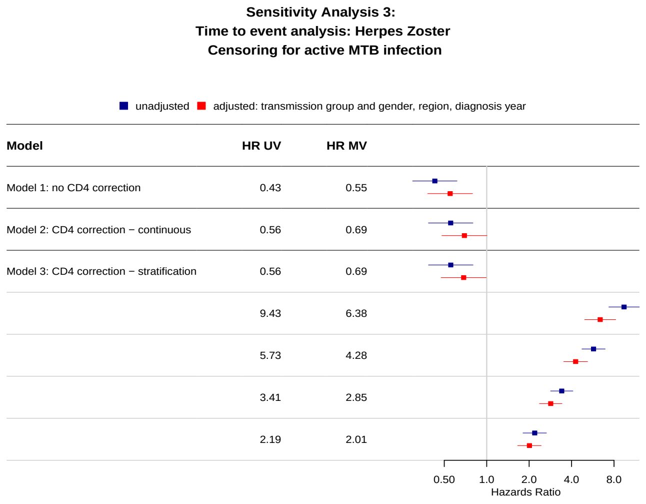

Fig BA. Time to event analysis of the occurrence of herpes zoster: Patients with tuberculosis infection compared to tuberculosis uninfected patients.

7 Alternative definitions for HIV set point virus load

The HIV set point virus load was defined as the mean of all ART-naive samples in the chronic phase of the patient. The duration of the chronic phase of the patients can vary, in addition to different time points for ART start. Moreover, the number of available samples per patient varied between 1 and 52 samples. To account for these differences, we modified the definition of HIV set point virus load in three ways:

1. *Alternative definition 1:* We excluded patients who either only had 1 rna sample or samples with a large variability (i.e., standard deviation > 1).
2. *Alternative definition 2:* We only included ART-naive, chronic samples taken within the first two years after HIV diagnosis.
3. *Alternative definition 3:* We only included ART-naive, chronic samples taken within the first two years after HIV diagnosis and excluded patients who either only had 1 rna sample or samples with a large variability (i.e., standard deviation > 1) in these two years.

	Original defintion	Alternative definition 1	Alternative definition 2	Alternative definition 3
Sample size	4516	2947	3706	2370
Overall mean:	4.4	4.3	4.35	4.23
Mean: No tuberculosis	4.43	4.33	4.37	4.26
Mean: LTBI	4.11	4.07	4.04	3.98
Mean: Active MTB infection	4.63	4.46	4.6	4.34
unadjusted: LTBI	-0.32 [-0.4, -0.24]	-0.25 [-0.33, -0.17]	-0.34 [-0.43, -0.25]	-0.28 [-0.38, -0.19]
unadjusted: active MTB infection	0.2 [0.03, 0.37]	0.13 [-0.08, 0.35]	0.23 [0.03, 0.42]	0.08 [-0.17, 0.33]
adjusted: LTBI	-0.21 [-0.28, -0.13]	-0.18 [-0.26, -0.1]	-0.23 [-0.32, -0.15]	-0.22 [-0.31, -0.12]
adjusted: active MTB infection	0.14 [-0.02, 0.31]	0.11 [-0.1, 0.31]	0.17 [-0.02, 0.36]	0.05 [-0.2, 0.29]

In the adjusted model, we corrected for the HIV risk group and gender, region, diagnosis year and CD4 nadir.

8 Summary of the sensitivity analyses

8.1 Set point virus load

Impact of LTBI on HIV set point virus load

UV MV

	OR UV	p UV	OR MV	p MV
Main Analysis	−0.32	< 0.0001	−0.21	< 0.0001
Def 2: Exclude patients with sd > 1	−0.25	< 0.0001	−0.18	< 0.0001
Def 3: Restricting to the first two years after HIV diagnosis	−0.34	< 0.0001	−0.23	< 0.0001
Def 4: Def 3 + Def 2	−0.28	< 0.0001	−0.22	< 0.0001
All patients with at least one test	−0.29	< 0.0001	−0.19	< 0.0001
Excluding patients with ambiguous test results	−0.32	< 0.0001	−0.22	< 0.0001
Restricting to patients with CD4 > 350	−0.3	< 0.0001	−0.23	< 0.0001
Restricting to patients with CD4 > 500	−0.29	< 0.0001	−0.24	< 0.0001
Correcting for CD4 at TB test date	−0.32	< 0.0001	−0.21	< 0.0001
TB test within 1 year of SHCS registration	−0.34	< 0.0001	−0.23	< 0.0001
Excluding patients with prophylactic treatment	−0.3	< 0.0001	−0.21	< 0.0001
Restricting to patients with subtype B	−0.36	< 0.0001	−0.26	< 0.0001
Correction for ethnicity	−0.32	< 0.0001	−0.2	< 0.0001
Restricting to white patients	−0.33	< 0.0001	−0.26	< 0.0001
Restricting to patients from Western Europe	−0.31	< 0.0001	−0.22	< 0.0001

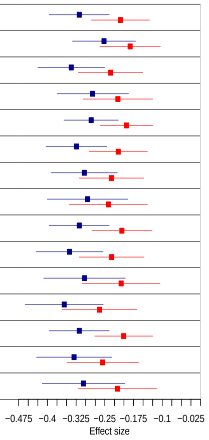

Fig BB. Summary: Association of LTBI with HIV set point virus load

8.2 Candida stomatitis

Candida stomatitis: Association with LTBI in the sensitivity analyses

UV MV

	OR UV	p UV	OR MV	p MV
Original Analysis	0.28	< 0.0001	0.68	0.0035
Including all patients	0.31	< 0.0001	0.66	0.0003
Exclude patients with ambiguous test results	0.28	< 0.0001	0.67	0.0035
Restricting to patients with CD4 > 350	0.38	< 0.0001	0.63	0.0090
Restricting to patients with CD4 > 500	0.42	< 0.0001	0.63	0.0339
Correcting for CD4 count at TB test date	0.28	< 0.0001	0.62	0.0003
TB test within 1 year of SHCS registration	0.28	< 0.0001	0.65	0.0090
Opportunistic infection within 2 years of TB test	0.28	< 0.0001	0.67	0.0155
Excluding patients with prophylactic treatment	0.3	< 0.0001	0.64	0.0148
Restricting to patients with subtype B	0.36	< 0.0001	0.67	0.0280
Correction for ethnicity	0.28	< 0.0001	0.67	0.0033
Restricting to white patients	0.41	< 0.0001	0.74	0.0520
Restricting to patients from Western Europe	0.35	< 0.0001	0.64	0.0100

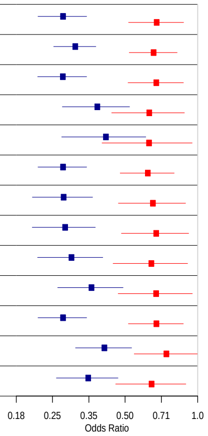

Fig BC. Summary: Association of LTBI and candida stomatitis.

8.3 Oral hairy leukoplakia

Oral hairy leukoplakia: Association with LTBI in the sensitivity analyses

■ UV ■ MV

	OR UV	p UV	OR MV	p MV
Original Analysis	0.28	< 0.0001	0.67	0.0330
Including all patients	0.36	< 0.0001	0.76	0.0789
Exclude patients with ambiguous test results	0.28	< 0.0001	0.68	0.0359
Restricting to patients with CD4 > 350	0.42	0.0001	0.74	0.2040
Restricting to patients with CD4 > 500	0.41	0.0020	0.62	0.1114
Correcting for CD4 count at TB test date	0.28	< 0.0001	0.65	0.0216
TB test within 1 year of SHCS registration	0.4	< 0.0001	0.92	0.6948
Opportunistic infection within 2 years of TB test	0.21	< 0.0001	0.49	0.0060
Excluding patients with prophylactic treatment	0.25	< 0.0001	0.52	0.0135
Restricting to patients with subtype B	0.28	< 0.0001	0.47	0.0034
Correction for ethnicity	0.28	< 0.0001	0.68	0.0408
Restricting to white patients	0.43	< 0.0001	0.76	0.1691
Restricting to patients from Western Europe	0.35	< 0.0001	0.63	0.0459

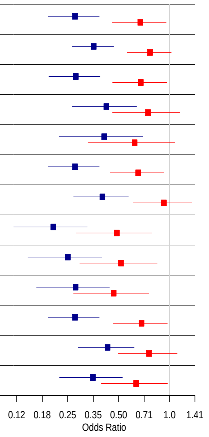

Fig BD. Summary: Association of LTBI and oral hairy leukoplakia.

8.4 Herpes zoster

Herpes zoster: Association with LTBI in the sensitivity analyses

■ UV ■ MV

	OR UV	p UV	OR MV	p MV
Original Analysis	0.47	< 0.0001	0.76	0.0844
Including all patients	0.57	< 0.0001	0.86	0.2513
Exclude patients with ambiguous test results	0.47	< 0.0001	0.75	0.0745
Restricting to patients with CD4 > 350	0.58	0.0036	0.81	0.2618
Restricting to patients with CD4 > 500	0.49	0.0037	0.61	0.0539
Correcting for CD4 count at TB test date	0.47	< 0.0001	0.68	0.0163
TB test within 1 year of SHCS registration	0.58	0.0014	0.88	0.4620
Opportunistic infection within 2 years of TB test	0.45	0.0004	0.65	0.0598
Excluding patients with prophylactic treatment	0.44	0.0001	0.69	0.0960
Restricting to patients with subtype B	0.38	< 0.0001	0.59	0.0300
Correction for ethnicity	0.47	< 0.0001	0.78	0.1071
Restricting to white patients	0.49	0.0003	0.71	0.1017
Restricting to patients from Western Europe	0.45	0.0004	0.67	0.0761

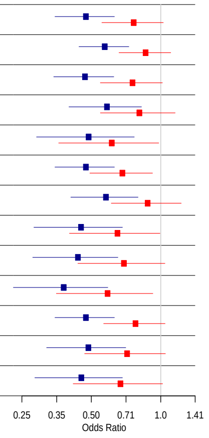

Fig BE. Summary: Association of LTBI and herpes zoster.
