# Supplementary material for: Diagnosis of latent tuberculosis infection is associated with reduced HIV viral load and lower risk for opportunistic infections in people living with HIV
Source: PLoS Biol. 2020 Dec 7;18(12):e3000963. doi: 10.1371/journal.pbio.3000963 (PMC7721132; doi:10.1371/journal.pbio.3000963)
Supplement: Attachment — Submitted filename: R1_PointByPoint_KusejkoNemeth.docx [file pbio.3000963.s006.docx]

**Point-by-point reply**

**Reviewer #1:**

**R1.1:** General: This interesting study investigates how testing positive for latent M.tuberculosis infection (LTBI) in those co-infected with HIV affects the HIV viral load, and the acquisition of other co-infections/co-morbidities. 
There is an extensive literature on how M.tuberculosis alters the immune system, including reducing antigen presentation, and beneficial T cell function. Thus, it may not be surprising that patients with clinical TB might have much more marked alterations of their immune system leading to a change in how HIV is controlled. This Swiss cohort provides a valuable opportunity to investigate these interactions between latent and active TB and HIV viral load and the number of opportunistic infections acquired. It is striking that having studied over 13,000 patients, the HIV nadir was 70 in TB compared to 265 in those with LTBI, and that the rate of opportunistic infections was lower in those with latent Mtb infection and HIV infection compared to those without Mtb infection, although as expected those with TB disease and HIV infection had increased rates of opportunistic infections.

**Answer:** We thank the reviewer for appreciating our study and the positive feedback, and in particular for taking the time to review this manuscript.

**R1.2:** There is an extensive set of supplementary information containing 56 supplementary figures., only a few of which are referred to in the text.

**Answer:** We agree with the reviewer that the links from the main manuscript to the supplementary figures were not discussed in sufficient detail in the text. To address this point, we included a new Figure (Figure 2), which now gives an overview of the sensitivity analyses performed. The supplementary material was divided into 2 parts: One giving additional information about tuberculosis tests, etc.; the other part reporting the results of the sensitivity analyses. In addition to the overview given in Figure 2, we referred to the supplementary material at several places throughout the manuscript.

Methods:

**R1.3:** The patients from the cohort all had positive or negative tests for LTBI; any patients with inconsistent results were excluded. This is a pity, as there is recent interest in those who convert to LTBI positive and then revert to negative. More information about the finding that inclusion of these individuals did not greatly affect the findings might be included.

**Answer:** We agree that patients who clear the TB infection or get newly infected might form an interesting population. However, we decided to exclude these patients as this might be a biased sub population: Over 80% (11057/13675) of the patients had only one tuberculosis test reported in the SHCS. We clarified in the Methods: “In the SHCS, one tuberculosis test is usually performed around study registration, further tests are not standard but all test results are recorded. In line with routine clinical practice and in line with all major clinical guidelines, most of the patients had only one LTBI test”. Hence, patients with more than one test form already a minority in our study population. Of the patients with more than one tuberculosis test reported in the SHCS, there were 187 patients with different results, i.e., at least one positive and at least one negative test (Figure 2 now includes a flow chart with all the numbers of the different study populations). Since we assumed the tuberculosis status to be “stable” over time, we excluded these patients from the main analysis (the assumption of a "stable" condition was tested in a different set of sensitivity analyses, see Figure 2). However, as suggested by the reviewer, we performed a new sensitivity analysis (see Appendix B1.1), where we included these 187 in the group of patients with latent tuberculosis infection. The results are similar to what we found in the main analysis. As an additional way to understand the impact of multiple TB tests, we also included a new sensitivity analysis where we only included patients who had a TB test within one year of SHCS registration, and used these test results for the categories disregarding later TB tests (see Section B2.1). Again, the stability of our results strengthens our main outcome.

Results:

**R1.4:** The text should be checked to ensure that when the LTBI group are described as having significant decreases in HIV viral load or opportunistic infections etc, it is clear whether it is the Mtb uninfected HIV infected group that the LTBI group is being compared to, or the TB patients group.

**Answer:** We thank the reviewer for this comment: In all our analyses, the group of MTB uninfected patients is the reference group, i.e., LTBI patients are always compared to MTB uninfected patients. We clarified this throughout the manuscript. For example, in the Methods: “In the first analysis, the association between MTB status (LTBI, active TB, or uninfected) and HIV SPVL was investigated using linear regression, with TB uninfected being the reference group.”

**R1.5:** LTBI is not a clear-cut state but rather a spectrum, as until TB is actually diagnosed, someone with incipient or subclinical TB will be categorised as having LTBI. There will also be individuals who test positive for Mtb infection using TST or an IGRA test, who then revert to negative, perhaps because the mycobacteria have been cleared. Overall, there will be fewer live mycobacteria in LTBI than in active TB disease, and as Mtb has the capacity to modulate immunity, it is perhaps less surprising that therefore patients with TB disease will have a more suppressed immune system and therefore be more susceptible to opportunistic infections.

**Answer:** We agree that the definition of LTBI would in the ideal case not be binary (latent vs. active). This is discussed as limitations and addressed with various sensitivity analyses: In particular, we used several approaches to understand the impact of our definition of LTBI, i.e., positive TB test but no clinically diagnosed TB. First, we performed extensive sensitivity analyses on the sensitivity and specificity of TB tests: As mentioned above, patients with different results over time were excluded (and included in a sensitivity analysis, B1.1), patients with ambiguous test results (borderline, or TST and IGRA having different results) were excluded (B1.2); the CD4 count at the time of LTBI test was further used to understand the impact of the immune status when performing a LTBI test (B1.3). All sensitivity analyses yielded robust results. In addition, we performed a time to event analysis where we pooled latent and active TB (B6): Here we performed a time-to-event analysis and censored for the development of active TB, to exactly address the problem of developing disease (B6). Again, our results are robust. In addition, we would like to highlight our new Figure 1 illustrating the timing of the studied events – regarding the assumption of LTBI being a stable state, please continue reading with the next point (R1.6)

**R1.6:** Secondly, if these are LTBI patients who have not progressed to active TB, due to the long follow-up of participants in this cohort, they will not be typical of LTBI individuals as a whole, as they are those with the ability to prevent progression to active disease, even when HIV-induced immunosuppression is present. In other words they may be a group protected against opportunistic infections as they have not progressed to TB. Assuming that the status "LTBI" is a stable condition, as discussed on page 13 (Discussion) is therefore a potential limitation of the approach taken. It may also be worth noting that progression from LTBI to active TB is likely to occur in the first 1-2 years after infection in about half of those infected who will progress to TB disease; thus if many of this cohort have been infected before coming to Switzerland, again most of those likely to progress to TB shortly after infection will have done so.

**Answer:** At this point, we would like to highlight the new Figure 1 included in this manuscript illustrating the timing of the studied events: the ART-naïve RNA measurements used to calculate SPVL as well as the diagnosis of opportunistic infections were close to the tuberculosis test date. Hence, the assumption of using “LTBI” as a stable condition is not such a limitation given the proximity of the events. To further understand the impact of this assumption, we performed a new sensitivity analysis looking at the timing of the events: 1) We included only patients who had a tuberculosis test within 1 year of the SHCS registration date and 2) we included only opportunistic infections within 2 years of the tuberculosis test: in the cross-sectional analysis as well as in the time-to-event analysis (censoring 2 years after the TB test). The obtained results are comparable to the ones presented in the main manuscript (see Appendix B2).

We agree with the reviewer that LTBI patients who do not progress to active TB might form a “special” group of patients: they are able to prevent progression to active diseases as well as might be able to have a better control with respect to HIV (SPVL) and opportunistic infections. This aspect is mentioned in the Discussion: “Causation of an observation is impossible to prove. We cannot rule out that the beneficial effects observed for patients with LTBI are due to host-specific factors that protect against active TB and other opportunistic infections and simultaneously improve control of HIV. Therefore, untested features of the innate immune system (e.g. macrophages (26)) could explain the association between LTBI and lower HIV SPVL. However, this explanation would require that those patients also maintain a more robust MTB specific CD4 T cell response in peripheral blood.”

**R1.7:** A statement might also be included about whether any of the individuals with LTBI were offered chemoprophylaxis.

**Answer:** There is a chapter about chemoprophylaxis in the supplementary material – again, we apologize that the link between the main manuscript and the supplementary material was not sufficient. With the new Figure 2, there should be a good overview of the different sensitivity analyses performed. Of the 840 patients classified as “LTBI”, 406 (48.3%) obtained prophylactic treatment (Rifampicin or Isoniazid). We performed a sensitivity analysis excluding these patients, which did not affect our main results, i.e., still significant reduction of HIV set point viral load and less opportunistic infections.

Minor comments:

**R1.8:** Abstract, third paragraph. TB infection was associated with a reduced HIV set point. The text should clarify if this is compared to those without Mtb infection, or those who developed active TB.
**Answer:** We apologize for not having been clear about this: In all analyses, the reference group was TB-uninfected patients; we clarified this throughout, e.g. in the abstract: “Compared to MTB uninfected patients, LTBI was associated with a reduced HIV set point viral load (hazard ratio = 0.24, p < 0.0001) in the adjusted model.”

**R1.9:** Abstract line 1, change "were ",  to "have been " exposed….Also, page 4, line 4.

**Answer:** We changed this as suggested.

**R1.10:** Page 9, line 2, change to "Similarly.."
**Answer:** We changed this as suggested.

_________________________________________________________________________________

**Reviewer #2:**

**R2.1:** General: This is an interesting paper which uses data from the Swiss HIV cohort to examine the association between latent TB infection (LTBI) and HIV clinical progression, including set-point viral load (VL) and the incidence of opportunistic infections (OIs). It purports to show that HIV+ patients with LTBI have a lower VL and lower incidence of OIs than HIV+ patients uninfected with TB, while there was evidence that patients with active TB disease have higher VL and incidence of OIs.

**Answer:** We thank the reviewer for appreciating our study and for taking the time to give us in-depth feedback.

**R2.2:** Strengths of this paper are the very large sample size, extensive data on potential confounding factors and systematic follow-up. However, there are also some serious weaknesses. First, one of the primary outcomes, set-point VL, was only available in about a third of the patients, so there are serious concerns about potential selection bias.

**Answer:** Set point viral load was defined as the mean of all ART-naïve HIV RNA measurements during the chronic phase of the HIV infection (at least 90 days after diagnosis and before the onset of AIDS). Given that many patients were diagnosed in the AIDS phase, or started with ART immediately, it is inevitable that such a measure can only be determined for a subset of the study population. We think that having such a measure available for a third of the population is actually more than what we anticipated: Given the heterogeneous nature of the diseases and the incredible dynamic developments in treatment and diagnostics over the years, our ability to include one third of patients is actually a strength and not a weakness: since 2010, when immediate ART was recommended by most treatment guidelines, no natural history of HIV can really be studied longitudinally anymore. We chose the HIV set point viral load as a measure which shows how well individuals can control HIV, before starting ART. We included a table in Appendix A3 comparing the groups of patients included in the SPVL analysis and those who were not included: One can see here that patients without SPVL were mostly diagnosed very late: the first CD4 count measured in the SHCS was on median 276, interquartile range = [120, 470], and the CD4 nadir was on median 140 [37, 280]. Hence, for most of the individuals without set point viral load there was no RNA measurement during the chronic phase available, most of them were in the AIDS-phase.

**R2.3:** There is also quite a high rate of losses to follow-up (over 20% of patients were lost).

**Answer:** We agree that loss to follow up might be problematic in the analysis of the occurrence of opportunistic infections: patients could develop such an infection after drop out. For this reason, we performed the time-to-event analysis for the 3 most frequent opportunistic infections. In this analysis, patients were censored after drop-out. Moreover, we would like to highlight the new Figure 1 which illustrates the timing of the events: the RNA measurements used to calculate HIV SPVL were very close to the LTBI test and SHCS registration date – for the analysis of HIV SPVL drop-out is therefore a minor problem. Moreover, most opportunistic infections were also diagnosed close to the registration date: To further assess the problem of loss to follow-up in the analysis of the occurrence of opportunistic infections, we included a sensitivity analysis following all patients only until 2 years after SHCS registration – the results are robust (see Appendix B2).

In general, it should be recognized that the loss to follow up percentage in the SHCS is quite low over the 30 years: Between 2-3 percent per year. This is remarkable for a longitudinal study of this size and representativeness, we are not aware of any study of this kind alike.

**R2.4:** ART would obviously be an important covariate but there is no information on how many patients were on ART at different times, or how this was incorporated in the analysis.

**Answer:** For the analysis of HIV set point viral load, only ART naïve samples were included, hence ART does not interfere with this analysis. For the analysis of opportunistic infections, again we would like to highlight the new Figure 1 about the timing of the different events (tuberculosis test, RNA measurement, diagnosis of opportunistic infection): almost all opportunistic infections were diagnosed around the SHCS registration date, i.e., before or shortly after starting with ART. However, to fully understand the potential impact of ART, we included a new sensitivity analysis: We performed a time-to-event analysis for the three most frequent opportunistic infections. We included only ART-naïve patients and censored for the start of ART. For all three diseases, we obtained significantly reduced hazards in the LTBI group as compared to the TB-uninfected group (see Appendix B3).

**R2.5:** The time sequence of events is also unclear. It seems that the classification of TB infection status is based on TB tests carried out at different times following diagnosis (and enrolment in the cohort), so some of these tests may have been conducted after the outcomes (VL and OIs) were recorded. This is also a problem for the analysis of active TB. Some more detailed comments are given below.

**Answer:** We thank the reviewer for this comment. We agree that the timing of the different events was not discussed well in the first version. We included a new Figure 1 to illustrate the timing of the different events (tuberculosis test, RNA measurement, diagnosis of opportunistic infection). In particular, Figure 1B shows how long before or after the tuberculosis test of the patient the opportunistic infections were diagnosed. Almost all diagnoses happened within 2 years of the tuberculosis test. We performed two new sensitivity analyses to understand the impact of the timing: 1) We included only patients who had a tuberculosis test within 1 year of the SHCS registration date and 2) we included only opportunistic infections within 2 years of the tuberculosis test: in the cross-sectional analysis as well as in the time-to-event analysis (censoring 2 years after the TB test). The obtained results are comparable to the ones presented in the main manuscript (see Appendix B2).

Major comments:

**R2.6:** The Abstract reports a reduced viral load as a "hazard ratio". This can't be correct?

**Answer:** We thank the reviewer for spotting this mistake, indeed, this is the regression coefficient. We rewrote this sentence accordingly: “Compared to MTB uninfected patients, LTBI was associated with a decrease of 0.24 of HIV set point viral load in the adjusted model (p < 0.0001).”

**R2.7:** The main paper needs to give clearer information about the test schedule for TB infection in the cohort. Information on this is given in the Supplementary Material, but understanding the timing of these tests, and the reasons for testing, seem important to interpret the results.

**Answer:** We fully agree with the reviewer and extensively added information about the timing of the tuberculosis test. In particular, we included the following in the Methods: “In the SHCS, one tuberculosis test is usually performed around study registration, further tests are not standard but all test results are recorded. In line with routine clinical practice and in line with all major clinical guidelines, most of the patients had only one LTBI test”. and the results: “In total, we analyzed test results from 17’243 different time points of 13’675 patients, with 11’057 (80.1%) patients having only one test available.” The reason for additional testing is not reported in the SHCS, hence the population of patients with more than one test result available might be biased. We performed a sensitivity analysis including only those patients having a test result within one year of SHCS registration (Appendix B2). We included a new Figure 1 illustrating the timing of all events, i.e., the time between the tuberculosis test and the HIV RNA measurements used to determine SPVL, and the time between the diagnosis of the opportunistic infection and the tuberculosis test. Finally, to provide a better overview over the different sensitivity analyses performed in Appendix B, we included a new Figure 2 illustrating the different analyses and sizes of the study populations.

**R2.8**: Later development of active TB is an exclusion criteria for the LTBI group. This will clearly impose a selection effect on this group so that those with more immune dysfunction are omitted. What implications might this have?
**Answer:** Our main study hypothesis was that patients with LTBI have heterologous protection against other infections, i.e., opportunistic infections and control of HIV. Development of active TB, however, weakens the immune system leading to more opportunistic infections as compared to MTB uninfected patients. Because of this, the strict distinction between “LTBI” and “active TB” was necessary in our study design. We agree with the reviewer that assuming these “stable” conditions over time might be problematic – most of the patients who develop active TB were latently infected before the outbreak of the disease. To understand the impact of the choice of our definition, we performed a sensitivity analysis pooling all TB infected patients and censoring for the diagnosis of active TB (see Appendix B6) and the results were robust. Thus, the selection effect mentioned by the reviewer cannot explain the observed protective effect of LTBI. We hope that this addresses exactly the concern of the reviewer.

**R2.9:** There are major concerns around the time sequence of events and therefore the direction of causality. A decline in immune function following infection may lead to the LTBI test becoming negative, especially in those with a high viral load and this might induce an apparent association between LTBI negativity and high viral load. This is a particular problem if LTBI test results are used from after the time the VL measurements are made. Maybe the analysis should be restricted to LTBI results taken within a short period after diagnosis?

**Answer:** As mentioned above, following this suggestion, we performed 2 sensitivity analyses (Appendix B2) to understand the impact of the timing: 1) We included only patients who had a tuberculosis test within 1 year of the SHCS registration and 2) we included only opportunistic infections within 2 years of the tuberculosis test. Again, we would like to emphasize the new Figure 1 which illustrates that the tuberculosis test, the diagnosis of the opportunistic infections and the RNA measurements cluster around the SHCS registration date. We thank the reviewer for this input, we think that the illustration of the timing of the events together with the two new sensitivity analyses strengthen our main conclusion. In addition to the above, we would also like to highlight that there are two sensitivity analyses (Appendix B1.3) regarding the CD4 count at the time of the tuberculosis test (>350 and >500, respectively), i.e., considering that tuberculosis test can become negative for declining immune function. The new Figure 2 now serves as a guide about the different analyses performed in Appendix B.

**R2.10:** As noted above, there are similar concerns with the analysis of OIs. In many cases, the LTBI test was done long after the occurrence of the OIs.

**Answer:** As mentioned above (R2.9), Figure 1B illustrates the time gap between the diagnosis of the opportunistic infection and the tuberculosis test. In the new sensitivity analysis, we included only opportunistic infections within 2 years of the tuberculosis test: in the cross-sectional analysis as well as in the time-to-event analysis (censoring 2 years after the TB test).

**R2.11:** I would have found it useful to have a diagram of the cohort showing the timing of the measurements used in this analysis.

**Answer:** As suggested, we included a new Figure 1 illustrating the timing of the events: 1A the time between the (ART-naïve) RNA measurements and the tuberculosis test, and 1B the time between the diagnosis of the opportunistic infection and the tuberculosis test.

**R2.12:** A key point is that ART status does not seem to be taken account of in this study. I assume that ART must have been in common use during the latter part of the cohort follow-up and may have had a major impact on TB infection status and on the outcomes of interest in this analysis.

**Answer:** We already answered to this point above (R2.4), but to summarize: 1) For the analysis of HIV set point viral load, only ART naïve samples were included, hence ART does not interfere with this analysis. 2) For the analysis of opportunistic infections, Figure 1B shows that almost all opportunistic infections were diagnosed around the SHCS registration date, i.e., before or shortly after starting with ART. We included a table in Appendix A3 comparing the groups of patients included in the SPVL analysis and those who were not included. In addition, we performed a time-to-event analysis for the 3 most frequent opportunistic infections including only ART-naïve patients and censored for the start of ART (Appendix B3).

**R2.13:** Viral load set-point data were only available in about a third of the patients and this could be a major source of selection bias. Did you look at the characteristics of those with and without VL data, to assess the likely degree of bias?

**Answer:** We thank the reviewer for this suggestion: We included tables in Appendix A3 comparing the groups of patients included in the SPVL analysis and those who were not included: One can see here that patients without SPVL were mostly diagnosed very late: the first CD4 count measured in the SHCS was on median 276, interquartile range = [120, 470], and the CD4 nadir was on median 140 [37, 280]. Hence, for most of the individuals without set point viral load there was no RNA measurement during the chronic phase available, most of them were in the AIDS-phase. We agree that we lose a lot of patients, however, we still think that HIV set point viral load is the ideal measure of determining how individuals can control HIV. This measure (set point virus load) has been shown to correlate with disease progression, and was used to study host and virus genetic effects on the course of disease extensively (Fellay, Science, 2007, doi: 10.1126/science.1143767; Bachmann, Retrovirology, 2017, doi: 10.1186/s12977-017-0356-3; Blanquart, Plos Biology, 2017, doi: 10.1371/journal.pbio.2001855).

**R2.14:** In the analyses of OIs, the adjusted effects are considerably smaller than the crude effects and some of the CIs are close to 1. This raises concerns over residual confounding, for example by features of immune status that are not fully captured by the CD4 count, or due to the limited frequency of CD4 testing.
**Answer**: We agree that this is a potential limitation and added the following text to the limitation section of our manuscript “However, it cannot be excluded that the results found in then multivariable model are due to residual confounding (most notably for Herpes Zoster)”. However, we would like to highlight that this was not a concern for the analysis of HIV set point virus load. In the case of opportunistic infections, the effects are still substantial (HRs <= 0.7 for LTBI) in the multivariable models, and these effects are consistent for the three OIs we investigated further (Candida stomatitis, Oral hairy leucoplakia and Herpes Zoster). Moreover, CD4 cell count was included in two different ways in the time-updated model: as continuous factor as well as categorical variable. Finally, the stability of our results in the large number of sensitivity analyses further strengthens the main study outcome.

Minor comments:

**R2.15:** TB is of course a major OI in its own right, but it is never stated that the analysis of OIs excluded pulmonary or extra-pulmonary TB.

**Answer:** We thank the reviewer for this comment: we included the information at several points in the Manuscript, e.g., in the Methods: “We tested the ten most frequent OIs diagnosed in the study population, excluding pulmonary or extrapulmonary tuberculosis”. We want to highlight that due to the heterogeneity of the diseases, we did not perform any analysis pooling opportunistic infections, i.e., active TB was only excluded for our choice of studied OIs.

**R2.16**: In the Methods, there is some text about MTB test results, concerning "skin diameter" and "immune status" that are obscure.

**Answer**: As suggested, this part was rewritten and now reads: “LTBI was defined as a positive skin reactivity test or positive IGRA and no development to active TB during follow up. The MTB test results were obtained in the form of P (positive), N (negative) and B (borderline) entries, i.e., the interpretation of the test results was performed by the treating physicians.”

**R2.17**: There is too much emphasis on arbitrary levels of "statistical significance". For example a statement that an effect "vanished" after adjustment whereas in fact the point estimate was still quite substantially below 1 (but the CI just overlapped 0).

**Answer:** We thank the reviewer for this input, we carefully worked over the statistical language of the paper.
 **R2.18:** The analysis of VL takes account of "nadir CD4 count" but there seems to be no limitation on the timing of that measure, which may have been a long time before or after the VL measurement (or the definition of TB infection status).

**Answer:** We thank the reviewer for this comment. We included a Figure in Appendix A5 illustrating that most of the CD4 nadir measurements were very close to the tuberculosis test, and hence – combining with the new Figure 1 in the main manuscript – also very close to the ART-naïve viral load measurements used to calculate HIV SPVL.

**R2.19:** You conclude that because the adjusted effect for active TB was close to zero that confounding must have been adequately controlled in the analysis for LBTI. That is quite a stretch as patterns of confounding may well differ between these different exposures.
**Answer:** We agree with the reviewer that factors influencing LTBI and active TB might differ. As suggested, we removed the corresponding statement.

**R2.20:** The treatment of LBTI as a "stable condition" is questionable given that LBTI results in HIV+ may well depend on the immune status of the individual, which can clearly vary over time.

**Answer:** To adjust for potential “false negative” tuberculosis test results, we performed sensitivity analyses restricting to patients with a CD4 count of at least 350 and 500 CD4 cells at the time of the tuberculosis test, respectively. The new Figure 2 gives an overview about all performed sensitivity analyses.

_________________________________________________________________________________
**Reviewer #3**

**R3.1:** Background. I'm not sure why we need to believe that human fire use sparked the evolution of Mtb!

**Answer:** As suggested, we modified the first sentence: “Mycobacterium tuberculosis (MTB) emerged as a human pathogen around 400.000 years ago.”, without mentioning the use of fire.

**R3.2:** The language is stilted.

**Answer:** We thank the reviewer for taking the time to read our manuscript in detail and for the feedback. A native speaker now helped us to improve the language.

**R3.3:** The word commensalism is not appropriate for Mtb, as we now know that the pathogen is likely to be in a constant stand-off with the immune system, rather than being simply a commensal. Low grade infection is also an imperfect representation. And it is not likely to be an interaction only with the innate immune system.

**Answer:** As suggested, we removed the word commensalism when talking about Mtb.

**R3.4:** The authors appear to suggest that active TB progresses HIV of its own right, driving HIV to progress. However, this misrepresents the fact that HIV must drive TB progression, as TB disease is far more common in HIV than HIV negative people in the same population. The hypothesis being test could be overtly and clearly stated.

**Answer:** We apologize that our study hypothesis was not clearly stated. The main aim of our study was to understand the impact of latent tuberculosis on the occurrence of opportunistic infections and HIV set point viral load. The inclusion of the group of patients who developed active tuberculosis served here rather as a control group: the hypothesis for this control group is that active tuberculosis further weakens the immune system, leading to more opportunistic infections and higher HIV set point viral load. The corresponding paragraph in the Introduction now reads: “In particular, we hypothesize that patients with LTBI have less opportunistic infections and can control HIV better as compared to MTB uninfected patients. The extension of this hypothesis predicts that active TB, which is associated with a pronounced inflammatory response and loss of the equilibrium between the host and the pathogen, reflects the breakdown of the protective state seen in LTBI and therefore is associated with more opportunistic infections (OIs) and faster progression of HIV infection.” The interplay of active TB and HIV was studied extensively in the past (e.g. Kwan CK, Ernst JD. HIV and tuberculosis: a deadly human syndemic. Clin Microbiol Rev. 2011 Apr;24(2):351–76) and was not our main focus, but it was nevertheless included mainly as a control, to test the opposite of our study hypothesis.

**R3.5:** Methods: These are presented soundly. Results: I think these are clearly presented.

**Answer:** We thank the reviewer for the appreciation of our methods and study results.

**R3.6:** Discussion: The concluding statement of a hypothesis that wasn't actually the hypothesis is an unfortunate ending to an otherwise solid discussion.

**Answer:** Again, we apologize for not having clearly stated our study hypothesis, we improved this throughout the manuscript. (see answer to R3.4 above)

**R3.7**: One consideration that isn't mentioned is that the LTBI positive individuals are a selective cohort in relation to TB, as those members that had progressive primary TB disease are excluded. In contrast, individuals who would have progressive primary disease upon infection are still included in the LTBI negative individuals. As such LTBI positive individuals are enriched for those capable of controlling Mtb. It would be reasonable to assume that they are able to control or repel other infections quite well too, including HIV.

**Answer:** Indeed, we cannot distinguish whether LTBI protects the patients or whether these patients have the ability of controlling TB as well as OIs and HIV. The corresponding paragraph in the Discussion reads: ”As with every observational study, causation of an observation is impossible to prove. Hence, in our study, we cannot rule out that the beneficial effects observed for patients with LTBI are due to host-specific factors protecting the individuals both from developing active TB and other opportunistic infections and simultaneously allow them controlling HIV better than other patients…” Independent of the underlying reason, the main hypothesis of our study is that patients with LTBI can control HIV better and have less OIs. Because of this, in the main manuscript, we grouped patients into “no TB”, “LTBI” and “active TB”. We included a new Figure 1 to highlight that all studied events (tuberculosis test, ART-naïve RNA measurements and diagnosis of opportunistic infections) cluster around the SHCS registration date. Because of this, the categories were assumed to be stable in our analysis. However, to understand the potential impact of individuals developing active TB while already being registered in the SHCS, we performed a sensitivity analysis of pooling all patients with TB (active and latent) and censored for progression to active tuberculosis. The main results – i.e., reduced hazards for developing opportunistic infections for patients latently infected with TB – could be reproduced in this analysis, strengthening our main message (Appendix B6), indicating that the enrichment-effect highlighted by the reviewer (i.e. that are enriched with those able to control TB and hence also other infections) cannot completely explain the effects of LTBI observed in this study.

**R3:** Tables and figures. These are well presented. 
**Answer:** Again, we would like to thank the reviewer for the appreciation of our study and for taking the time to give us feedback.

_________________________________________________________________________________

**Reviewer #4**:

**R4.1:** This is a well-written and absorbing manuscript which starts with the exciting hypothesis that acquisition of M.tb infection provides a degree of protection against other conditions in the context of HIV co-infection. The authors marshal helpful data to support this. They then go on to show that there are differences between an M.tb uninfected population (the great majority of the Swiss HIV Cohort Study group being examined), individuals with latent TB infection and those who develop active TB during follow up. This appears to be related to a reduced HIV set point viral load in the latent TB population. Further, they had lower odds of oral thrush and OHL compared to individuals who were considered to be M.tb-uninfected, or had active TB disease.
**Answer:** We thank the reviewer for appreciating our study and for taking the time to review our manuscript in detail.

**R4.2:** There may be something in it, but I am not sure that the authors have fully convinced me with the manuscript as it stands. One major issue is that as far as I can understand, the cohorts are defined by their status (i.e. M.tb-uninfected, M.tb-infected and TB disease) at some point in time, and assessments occur going forward from then. Given that the date of diagnoses varied between the three groups (as would be expected active TB was diagnosed at an earlier year to latent TB), and there appears to be little mention of antiretroviral therapy throughout the manuscript, I am struggling a bit with some of their findings.

**Answer:** We appreciate the concerns of the reviewer. Indeed, the timing is an important aspect which was not emphasized enough in the first version. The problem of the different time points of the events (HIV set point viral load, tuberculosis test, opportunistic infection) is actually not that problematic as it might seem: in fact, most of the tuberculosis tests were performed around the SHCS registration date, as was the diagnosis of an opportunistic infection and the (ART-naïve) RNA measurements for the calculation of HIV set point virus load. To illustrate the timing of the measurements considered for our analysis, we included a new Figure 1. Figure 1A shows the (ART- naïve) RNA measurements over time as well as the time points the measurements were taken (time gap between tuberculosis test and RNA measurement). Figure 1B shows the timing of the diagnoses of the opportunistic infections (time gap between diagnosis date and tuberculosis test). The vast majority of these events cluster around the SHCS registration date. Hence, there are rarely cases where the opportunistic infection was diagnosed years after the tuberculosis test. To further understand the impact of the timing, we performed 2 new sensitivity analyses: first, we restricted the population to all patients who had a tuberculosis test or active tuberculosis within 1 year of the SHCS registration date (and grouped the patients according to these results); second, we only considered opportunistic infection whose diagnosis date differed by less than 2 years from that of the tuberculosis test, and censored 2 years after tuberculosis test in the time-to-event analysis. The results were stable (Appendix B2), indicating that for our purpose, assuming the TB status to be stable over time is reasonable. We thank the reviewer for this input, we think that these additional analyses and consideration of timing further strengthen our results. Regarding ART: For the analysis of HIV set point viral load, only ART-naïve measurements were considered. For the analysis of opportunistic infection, we would again like to refer the reviewer to our new Figure 1B: the events cluster around the SHCS registration date (and tuberculosis test), hence most individuals were not on ART or only recently started. To further understand the impact of ART in the analysis of OIs, we included a new sensitivity analysis (Appendix B3): in the time-to-event analysis of the three most frequent OIs, we only included ART-naïve patients and censored at the start of ART. We still see significantly reduced hazards for OIs in the group of LTBI patients as compared to TB-uninfected patients.

**R4.3:** For example in the Methods, the authors state that "patients with positive and negative M.tb tests at different time points were excluded". Does this mean that they did or did not perform routine serial testing for latent TB infection? If they did, were those participants who were initially negative and subsequently tested positive not included in the study? If this is a case this feels like a shame as this population may provide insights into their proposed protection conferred by M.tb (for example were less OI events occurring after M.tb infection compared to before?). It feels like I am missing something here as this is a clear benefit of an observational cohort, which has the flexibility to asses an individual at different points throughout their health trajectory. Other comments (plus some further discussion of the above) are given below as they arise in the manuscript.

**Answer:** There is no routine serial testing for latent TB in the SHCS, we included this information now in the Methods: “In the SHCS, one tuberculosis test is usually performed around study registration, further tests are not standard but all test results are recorded. In line with routine clinical practice and in line with all major clinical guidelines, most of the patients had only one LTBI test”. We included the following in the Results: “In total, we analyzed test results from 17’243 different time points of 13’675 patients, with 11’057 (80.1%) patients having only one test available.” Hence, the sub population of patients with more than one test is biased. Because of this, we decided to exclude patients with different results over time from our analysis. We would like to emphasize that this was the case for only 187 patients – see new Figure 2 for the numbers in the different subpopulations. We however performed a new sensitivity analysis including these patients again and the results we obtained were similar to the main results (see Appendix B1).
 
**R4.4:** Methods - Study population and definitions. I would be interested to know the number or proportion of participants who had multiple tests? And also those where there were negative then positive results or vice versa.

**Answer:** As suggested, we included in the main manuscript the number of patients with only one test (“In total, we analyzed test results from 17’243 different time points of 13’675 patients, with 11’057 (80.1%) patients having only one test available.”) and included a section in the supplementary material describing more detailed the number of LTBI tests (see Appendix A, Figure A3). Moreover, we included a flow chart in the main manuscript, Figure 2, describing the numbers in the different study population in the sensitivity analyses, i.e., one can now also see in the main manuscript that only 187 patients were excluded (positive and negative tuberculosis test results at different time points). Of these 187 patients, 94 had at least one positive TB test followed by at least one negative TB test, 86 patients vice versa; and 7 patients had alternating results.

**R4.5:** Methods - The authors mention that they used all TB test results including tests performed before, during or after entry to the Swiss HIV cohort study. Can they provide any information on the proportions of these? This might be important if there were a particularly large group diagnosed prior to the Swiss HIV cohort, as there could be concerns about possibly self-reported or non-observed test results.

**Answer:** As suggested, we provided more information about the timing of the tuberculosis test. In the main manuscript, we included a new Figure 1 where we show the number of viral load measurements and diagnoses of opportunistic infections over time – always looking at the time between the lab/diagnosis date and the first tuberculosis test of the patients. In addition, we added a section in Appendix A1: “Of the 17 243 tuberculosis test dates, 5750 (33.3%) tuberculosis tests were at date of the SHCS registration, 10 146 (58.8%) tests were within 1 year of the SHCS registration date, 182 (1.1%) more than 1 year before the SHCS registration and 6915 (40.1%) more than one year after SHCS registration.” Hence, the vast majority of tuberculosis tests were close to the SHCS registration date or after, effectively eliminating incorrect reports of previous test results as a serious source of error. Also, all of the results entered retrospectively to the study were taken from the medical records of the patients, not based on self-reported information. In addition to this descriptive information, we also performed a new sensitivity analysis where we only included patients who had the tuberculosis test or clinically diagnosed tuberculosis within one year of the SHCS registration (see Appendix B2). The results are stable to this restriction of the study population.

**R4.6:** Methods - The authors are keen on HIV set point viral load. This is certainly a useful measure, but I am concerned that its value diminishes in the active TB population, when so many participants in the HIV cohort have presented with TB which then led to an HIV diagnosis, that only around 10% of cases are used in this analysis in the TB disease group. How do the authors answer this point?

**Answer:** HIV set point viral load was defined as the mean of all ART-naïve RNA measurements taken in the chronic phase of the disease, i.e., before the occurrence of opportunistic infections. This measure has been proven to be a good measure of how well individuals can control HIV. There is no other measure which is routinely available and could be used to study this – using single RNA measurements during ART or during the AIDS-phase would not provide a stable measure. We agree that one inevitable drawback of this measure is that we can only include this relatively small subset of patients: those with ART-naïve samples before the occurrence of opportunistic infections. However, we would like to emphasize that many individuals in the past were diagnosed with HIV when they already had opportunistic infections, and many individuals diagnosed in the later years started ART directly – so having set point viral load measurements for 4 516 is actually quite a high sample size. The focus of our study was to study latent TB, in particular comparing SPVL between LTBI and TB-uninfected patients. The inclusion of the group of patients who developed active TB served rather as a control group. In this analysis, we could only include patients who developed active TB later, leading to this low percentage of patients with available set point viral load.

**R4.7:** Methods - Statistical analysis. As mentioned above it would be useful to know what proportion of subjects have an initial negative skin or blood test for latent TB infection and then became positive. Assuming that this does reflect an immune response against M.tb, and samples were assessed at different time points, one might expect there to be the same effect as the authors report in their cross sectional analysis in specific individuals followed over time. Do they have any evidence to demonstrate this?

**Answer:** As mentioned above (R4.3), we included additional information about the patients with different tuberculosis test results over time: in total 187 patients. As the majority of patients only have one tuberculosis test, the population of patients with more than one test is likely to be biased. In addition, given the low number of patients with different test results over time (and different patterns over time), we refrain from performing more in-depth analysis on these patients. But we included a new sensitivity analysis including these 187 patients again (see Appendix B1) – the results are stable.

**R4.8:** Methods - Statistical analysis. The authors appear to shy away from mentioning antiretroviral therapy. I might have missed it but to me this is so fundamentally important to HIV care and reduction in opportunistic infections and also in controlling true pathogens such as M.tb, that if feels as if they are trying to bury something. Can the authors reassure me about this?

**Answer:** We thank the reviewer for this comment, indeed, discussion about antiretroviral therapy was missing in the manuscript. It was not our intention to shy away from this topic, but actually for our study question, antiretroviral therapy plays a minor role: For the analysis of HIV set point viral load, we only included HIV RNA measurements while the patients were still ART-naïve. By definition, ART does thus not interfere with this analysis. We would also highlight again at this point that we included a new Figure 1 showing the time line of the studied events: all events (tuberculosis tests, opportunistic infections, viral load measurements) used for our analyses were clustered around the SHCS registration date, hence most individuals were ART-naïve or started around the same time of the first diagnosis of an opportunistic infection. In the case of the analysis of opportunistic infections, we included a new sensitivity analysis: for the time-to-event analysis of the 3 most frequent opportunistic infections, we only followed ART-naïve patients and censored for the start of ART. The results are stable, i.e., for all 3 diseases, the group of LTBI patients had significantly reduced hazards (see Appendix B3).

**R4.9:** 6.      Results - As mentioned earlier I am concerned about the relative proportion of individuals who had latent TB compared to active TB. These are pretty much the same number. I think most clinicians would expect to see at least four times as many participants with latent TB than active disease. I appreciate that people can progress from latent infection to active disease, but then things might get messy with regards to where the participant is categorised i.e. as latent infection or active TB disease. Can the authors explain.

**Answer:** All individuals with a clinically diagnosed active TB were categorized as having “active TB”, regardless of other tuberculosis tests performed and regardless of the time point of this diagnosis. To justify using this “stable” definition of TB, we would like to highlight once more the new Figure 1, where we illustrated the timing of the events: All opportunistic infections (including active TB) as well as the tuberculosis test are quite close in time. Regarding the proportion of individuals with latent TB versus active TB, we agree that this is surprising and counterintuitive for clinicians and those familiar with the literature. However, the unique strength of the SHCS is the high quality of longitudinal follow up data with a very accurate clinical phenotype. Our intuition and most clinical evidence stem from short term observations and/or cross-sectional studies. Hence, it is possible that we miss a significant number of TB cases without the stringent follow-up and detailed clinical phenotypes over decades. One observation supportive of this hypothesis is the observation that in low resource settings, TB is often missed in patients with HIV infection (Bates, Lancet ID, 2015, doi: 10.1016/S1473 3099(15)70058-7).

**R4.10**: Results - The information for HIV set point viral load feels to me a bit shaky. The numbers of subjects where this information was available were quite reduced. Is there any evidence that we are looking at some form of selection bias? Again I would be interested to know how the authors have accounted for the use of antiretroviral therapy for an individual and also between the different groups.

**Answer**: HIV set point viral load was defined as the mean of all ART-naïve measurements during the chronic phase of the HIV infection (>90 days after diagnosis and before the occurrence of any opportunistic infections). Hence, all measurements were before the start of ART, i.e., no adjustment for ART is needed here – we apologize in case this was not clear in the first version, we clarified at several points now that only ART-naïve samples were considered. Many individuals were diagnosed with HIV after (or due to) the occurrence of opportunistic infections (especially in the early years) and many individuals started ART directly after diagnosis (especially later years). Hence, it is inevitably that we cannot determine the SPVL for many individuals. We would like to highlight that being able to study HIV set point viral load.- i.e., how well the individual can control HIV without ART – in almost a third of our patients, is already quite good. As suggested by the reviewer, we included a table in Appendix A3 comparing the population of patients with and those without set point viral load, also stratified by TB status. One can see here that patients without SPVL were mostly diagnosed late: the median of the first CD4 count measured in the SHCS was 276, interquartile range = [120, 470], and the median of the CD4 nadir was 140 [37, 280]. Hence, for most of the individuals without set point viral load there was no RNA measurement during the chronic phase available because most of them were in the AIDS-phase. We thank the reviewer for the feedback, this table helps understanding why HIV set point viral load could not be determined for so many patients.

**R4.11:** The Discussion is readable but feels far too long. It takes up five of the twelve total pages of manuscript in my version. I think this could be significantly reduced with no loss of impact.

**Answer:** As suggested by the reviewer, the discussion was rewritten and condensed at many parts.

**R4.12:** Tables and figures - Table 1 is quite large - I think the authors could collapse some of the sub groups with no loss of impact.

**Answer:** As suggested, Table 1 was shortened: In particular, detailed information about ethnicity, region and HIV subtype was moved to the supplementary material (Appendix A6).

**Figure 1**

**Figure 2**

*Figure 2: Description of the study population and the sensitivity analyses, B1 – B7 refer to the respective sections in the Supplementary Material B.*
