# Supplementary material for: Diagnosis of latent tuberculosis infection is associated with reduced HIV viral load and lower risk for opportunistic infections in people living with HIV
Source: PLoS Biol. 2020 Dec 7;18(12):e3000963. doi: 10.1371/journal.pbio.3000963 (PMC7721132; doi:10.1371/journal.pbio.3000963)
Supplement: Attachment — Submitted filename: PointByPoint.docx [file pbio.3000963.s007.docx]

**Point-by-point reply**

**Editorial comments**

1) Please restructure the main text according to PLOS Biology format, i.e. Abstract, Introduction, Results, Discussion and Methods. The figures need to be cited in order, and figure 1 is only cited in the Methods, so would either need to be cited at the beginning of the results (best possibility) or be moved to the last main display item.

**Answer:** The manuscript was re-structured and Figure 1 cited at the beginning of the results.

2) Please include the specific protocol number(s) that were approved by the ethics committees of the participating institutions, if available.

**Answer:** We adapted our ethics statement as follows: *“The SHCS was approved by the local ethical committees of the participating centers: Kantonale Ethikkommission Zürich (KEK-ZH-NR: EK-793); Ethikkommission beider Basel ("Die Ethikkommission beider Basel hat die Dokumente zur Studie zustimmend zur Kenntnis genommen und genehmigt."); Kantonale Ethikkommission Bern (21/88); Comité departmental d'éthique des specialités médicales es de médecine communautarie et de premier recours, Hôpitaux Universitaires de Genève (01–142); Commission cantonale d'éthique de la recherche sur l'être humain, Canton de Vaud (131/01); Comitato etico cantonale, Repubblica e Cantone Ticino (CE 813); Ethikkommission des Kantons St. Gallen (EKSG 12/003), and written informed consent was obtained from all participants.”*

3) In all figure legends please include statistical information, including how many times measurements were performed (and whether they were technical and biological replicates) and, where applicable, what is being represented (e.g. mean or median, plus confidence interval).

**Answer:** We expanded on the figure legends providing more information on what is presented, e.g., odds ratios and confidence intervals.

4) Please note that per journal policy, all of the individual quantitative observations that underlie the data summarized in the main and supplementary (i.e. those in the appendices) figures and tables of your paper need to be made available in one of the following forms:

a) Supplementary files (e.g., excel). Please ensure that all data files are uploaded as 'Supporting Information' and are invariably referred to (in the manuscript, figure legends, and the Description field when uploading your files) using the following format verbatim: S1 Data, S2 Data, etc. Multiple panels of a single or even several figures can be included as multiple sheets in one excel file that is saved using exactly the following convention: S1_Data.xlsx (using an underscore).

b) Deposition in a publicly available repository. Please also provide the accession code or a reviewer link so that we may view your data before publication.

Regardless of the method selected, please ensure that you provide the individual numerical values that underlie the summary data displayed throughout the study (note that we do not require all raw data, just the numerical data underlying the figures and tables), as they are essential for readers to assess your analysis and to reproduce it.

NOTE: the numerical data provided should include all replicates AND the way in which the plotted mean and errors were derived (it should not present only the mean/average values).

**Answer:** We included three excel files, for the main manuscript, for Appendix A and for Appendix B including all data underlying the Figures (using several sheets in the same excel file).

5) Please also ensure that figure legends in your manuscript include information on where the underlying data can be found, and ensure your supplemental data file/s has a legend.

**Answer:** We included information on where to find the numerical values in the figure legends.

6) Please ensure that you update your Data Statement in the submission system to accurately describe where your data can be found. That is, in addition to the restrictions and instructions to obtaining the raw data that you describe, please also state where the numerical data underlying the figures can be found.

**Answer:** We adapted the Data Statement accordingly.

------

**Response to reviewers**

**Reviewer remarks:**

**Reviewer #2:** I have now been able to review the revised version of this paper. I am pleased to say that I am satisfied with the changes that have been made as well as the response letter from the authors.

Just a few minor points to pass on to the authors for their consideration when finalizing the manuscript for publication:

1.      Figure 1a and 1b: The legend and labelling really need to be improved. In Fig 1a, what are the units of the dates (or rather differences of dates) on the x axis? Are they in years? What are the 3 lines marked on Fig 1a?

**Answer:** Figure 1: We included the legend and rephrased the x-axis label to “Years between HIV RNA date and date of the first tuberculosis test”. Figure 2: We rephrased the x-axis label to “Years between diagnosis date and date of the first tuberculosis test.” We apologize that we missed to include the legends in the earlier version of the manuscript.

2.      Foot of page 11. The reference to an "interaction between active TB and OIs": The term "interaction" has a specific statistical meaning which is not what is intended here. The word "association" would be clearer.

**Answer:** As suggested, we changed “interaction” to “association”.

3.      Foot of page 12. "…could be due to lower sample sizes" - I think they are actually referring to the small "numbers of events" rather than "small sample size".

**Answer:** We changed this as suggested.

4.      Abstract: I think the 0.24 decrease in viral load actually refers to the log viral load? Could this be clarified?

**Answer:** Indeed, we changed this to log viral load.

**Reviewer #4:** Thanks for the useful revisions.

Figure 1 is helpful, though I couldn't find legends that explain how the X axis timeline should be interpreted, and also indicated to which patient populations the colours  in the plots referred.

**Answer:** Figure 1: We included the legend and rephrased the x-axis label to “Years between HIV RNA date and date of the first tuberculosis test”. Figure 2: We rephrased the x-axis label to “Years between diagnosis date and date of the first tuberculosis test.” We apologize that we missed to include the legends in the earlier version of the manuscript.
